# *HNF4A* and *GATA6* Loss Reveals Therapeutically Actionable Subtypes in Pancreatic Cancer

**DOI:** 10.1016/j.celrep.2020.107625

**Published:** 2020-05-12

**Authors:** Holly Brunton, Giuseppina Caligiuri, Richard Cunningham, Rosie Upstill-Goddard, Ulla-Maja Bailey, Ian M. Garner, Craig Nourse, Stephan Dreyer, Marc Jones, Kim Moran-Jones, Derek W. Wright, Viola Paulus-Hock, Colin Nixon, Gemma Thomson, Nigel B. Jamieson, Grant A. McGregor, Lisa Evers, Colin J. McKay, Aditi Gulati, Rachel Brough, Ilirjana Bajrami, Stephen J. Pettitt, Michele L. Dziubinski, Simon T. Barry, Robert Grützmann, Robert Brown, Edward Curry, Glasgow Precision Oncology Laboratory, Australian Pancreatic Cancer Genome Initiative, Marina Pajic, Elizabeth A. Musgrove, Gloria M. Petersen, Emma Shanks, Alan Ashworth, Howard C. Crawford, Diane M. Simeone, Fieke E.M. Froeling, Christopher J. Lord, Debabrata Mukhopadhyay, Christian Pilarsky, Sean E. Grimmond, Jennifer P. Morton, Owen J. Sansom, David K. Chang, Peter J. Bailey, Andrew V. Biankin

**Affiliations:** 1 Institute of Cancer Sciences, University of Glasgow, Garscube Estate, Switchback Road, Bearsden, Glasgow G61 1QH, Scotland; 2 Cancer Research UK Beatson Institute, Garscube Estate, Switchback Road, Glasgow G61 1BD, UK; 3 Epigenetics Unit, Department of Surgery & Cancer, Imperial College London, Hammersmith Campus, Du Cane Road, London W12 0NN, UK; 4 West of Scotland Pancreatic Unit, Glasgow Royal Infirmary, Glasgow G31 2ER, UK; 5 Stratified Medicine Scotland Innovation Centre, Queen Elizabeth University Hospital, Glasgow G51 4TF, UK; 6 MRC–University of Glasgow Centre for Virus Research, University of Glasgow, Garscube Estate, Switchback Road, Bearsden, Glasgow G61 1QH, Scotland; 7 CRUK Gene Function Laboratory and Breast Cancer Now Toby Robins Research Centre, The Institute of Cancer Research, Fulham Road, London SW3 6JB, UK; 8 Department of Molecular and Integrative Physiology, University of Michigan, 4304 Rogel Cancer Center Drive, Ann Arbor, MI 48109, USA; 9 Bioscience, Oncology, IMED Biotech Unit, AstraZeneca, Cambridge, UK; 10 Department of Surgery, Universitätsklinikum Erlangen, Erlangen, Germany; 11 The Kinghorn Cancer Centre, 370 Victoria Street, Darlinghurst and Garvan Institute of Medical Research, Sydney, NSW 2010, Australia; 12 St Vincent’s Clinical School, Faculty of Medicine, University of New South Wales, Sydney, NSW, Australia; 13 Mayo Clinic, Rochester, MN 55905, USA; 14 UCSF Helen Diller Family Comprehensive Cancer Center, San Francisco, CA 94158, USA; 15 Pancreatic Cancer Center, Perlmutter Cancer Center, NYU Langone Health, New York, NY 10016, USA; 16 Cold Spring Harbor Laboratory, Cold Spring Harbor, NY, USA; 17 Department of Biochemistry and Molecular Biology, Mayo Clinic College of Medicine and Science, Jacksonville, FL 32224, USA; 18 University of Melbourne Centre for Cancer Research, University of Melbourne, Melbourne 3010, VIC, Australia; 19 South Western Sydney Clinical School, Faculty of Medicine, University of New South Wales, Sydney, NSW, Australia; 20 Department of General Surgery, University of Heidelberg, Heidelberg 69120, Germany; 21 These authors contributed equally; 22 Lead Contact

## Abstract

Pancreatic ductal adenocarcinoma (PDAC) can be divided into transcriptomic subtypes with two broad lineages referred to as classical (pancreatic) and squamous. We find that these two subtypes are driven by distinct metabolic phenotypes. Loss of genes that drive endodermal lineage specification, *HNF4A* and *GATA6*, switch metabolic profiles from classical (pancreatic) to predominantly squamous, with glycogen synthase kinase 3 beta (GSK3β) a key regulator of glycolysis. Pharmacological inhibition of GSK3b results in selective sensitivity in the squamous subtype; however, a subset of these squamous patient-derived cell lines (PDCLs) acquires rapid drug tolerance. Using chromatin accessibility maps, we demonstrate that the squamous subtype can be further classified using chromatin accessibility to predict responsiveness and tolerance to GSK3β inhibitors. Our findings demonstrate that distinct patterns of chromatin accessibility can be used to identify patient subgroups that are indistinguishable by gene expression profiles, highlighting the utility of chromatin-based biomarkers for patient selection in the treatment of PDAC.

## In Brief

Brunton et al. demonstrate that differential chromatin accessibility can predict responsiveness and tolerance to GSK3β inhibitors in the squamous subtype of PDAC. This study provides an important proof of concept that chromatin accessibility can be used to identify additional PDAC subgroups with potential therapeutic utility.

## Graphical Abstract

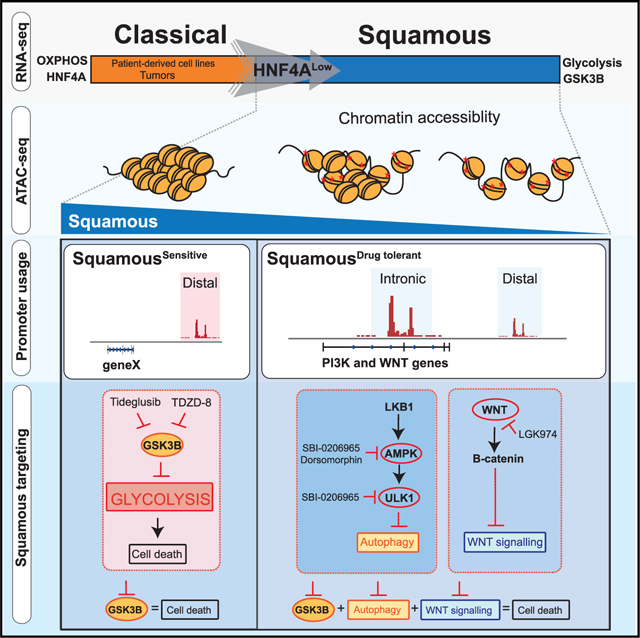

## INTRODUCTION

The prognosis for patients suffering from pancreatic ductal adenocarcinoma (PDAC) is extremely poor, with less than 8% of patients surviving for more than 5 years after diagnosis. PDAC is defined by a complex and heterogeneous mutational landscape with a handful of highly recurrent mutations in well-described cancer genes and a plethora of low-frequency events associated with genes of often unknown function (Bailey et al., 2016; Biankin et al., 2012; Humphris et al., 2017; Waddell et al., 2015; Witkiewicz et al., 2015). Establishing which of these events drive tumor progression and/or survival has proved challenging. One obstacle is our limited ability to stratify patients for targeted therapy and a lack of biomarkers to direct clinical decision-making (Biankin et al., 2015). Improved patient stratification and more effective approaches to therapy are urgently needed to improve outcomes for pancreatic cancer.

Recent integratomic studies have demonstrated that PDAC is composed of two major transcriptomic subtypes, namely, classical (pancreatic) and squamous, which are characterized by distinct mutations, gene expression profiles, and prognosis (Bailey et al., 2016; Collisson et al., 2011, 2019; Moffitt et al., 2015). The classical (pancreatic) subtype is characterized by differentiated duct cell marker expression and a favorable prognosis, whereas the squamous subtype is associated with gene silencing of endoderm specification genes, such as *HNF1A*, *HNF4A*, and *GATA6*; metabolic reprogramming; and poor clinical outcome. Importantly, the dynamic changes in gene expression observed between the classical (pancreatic) and squamous subtypes are driven by alterations in the epigenetic landscape (Bailey et al., 2016; Lomberk et al., 2018; Somerville et al., 2018). The squamous subtype is further typified by mutations in members of the COMPASS-like complex that regulate histone methylation, including *KDM6A*, *MLL2*, and *MLL3* (Andricovich et al., 2018; Bailey et al., 2016).

Gene programs that characterize PDAC squamous tumors include those involved in hypoxia response, metabolic reprogramming, and autophagy (Bailey et al., 2016), suggesting that metabolic targeting in this subtype may be effective. Extensive work by others has shown that metabolic rewiring is central to PDAC’s ability to survive within a nutrient- and oxygen-depleted tumor microenvironment (Chini et al., 2014; Commisso et al., 2013; Guillaumond et al., 2013; Son et al., 2013). Moreover, the major oncogenic driver in PDAC, *KRAS*, along with the selective pressure of a hypoxic tumor environment can promote metabolic rewiring through stimulating glycolysis (Ying et al., 2012) and autophagy (Yang and Kimmelman, 2011, 2014). These studies also highlight the intrinsic metabolic plasticity of pancreatic cancer cells, which may, in part, explain the lack of significant therapeutic benefit of metabolic targeting (Baek et al., 2014; Boudreau et al., 2016; Sancho et al., 2015). Furthermore, recent data now suggest that plasticity exists between subtypes. For example, the targeted inhibition of Colony-Stimulating Factor 1 Receptor (CSF1R) in LSL-Kras^G12D/+^;Trp53^fl/+^;Pdx1-Cre (KPC) genetically engineered mouse models (GEMMs) results in a profound reprogramming of tumor cell-intrinsic pathways from predominantly squamous to classical (pancreatic) (Candido et al., 2018). Likewise, MET Proto-Oncogene, Receptor Tyrosone Kinase (MET) inhibition in squamous PDAC induces a transcriptional switch toward classical (pancreatic) associated gene programs, in particular those driven by GATA6 (Lomberk et al., 2018). Therefore, metabolic plasticity or adaptation and therapy-induced subtype switching may represent important implications for disease progression, drug resistance, and the development of subtype-specific therapies. Deciphering the transcriptional regulatory networks underpinning subtype plasticity has the potential to identify therapeutic vulnerabilities and nodes of therapy evasion.

To address these questions, we used a set of 48 early-passage PDAC patient-derived cell lines (PDCLs) that provide an isogenic and experimentally tractable system for developing and validating subtype-dependent therapeutic vulnerabilities. We show that PDCLs recapitulate major metabolic transcriptional profiles observed in bulk PDAC tissue, and that plasticity exists between PDAC subtypes. Specifically, HNF4A and GATA6 loss in a classical (progenitor) background can drive a switch toward squamous-associated metabolic reprograming events and identify GSK3b as a driver of glycolysis. Pharmacological inhibition of GSK3b showed selective sensitivity in the squamous subtype; however, a subset of these squamous PDCLs acquire rapid drug tolerance. Using assay for transposase-accessible chromatin sequencing (ATAC-seq) analysis, we show that the squamous subtype separates into two distinct chromatin subgroups with unique chromatin accessibility and promoter usage. We demonstrate that the drug-tolerant squamous subgroup has access to an amplified WNT signaling program via application of both intronic and distal promoter usage. Using both transcriptomic and chromatin landscape profiles, we provide a model system to predict PDAC responders and non-responders to subtype-specific therapeutic vulnerabilities.

## RESULTS

### PDAC PDCLs Recapitulate Metabolic Profiles Observed in PDAC Bulk Tumor Tissue

**Figure 1.**
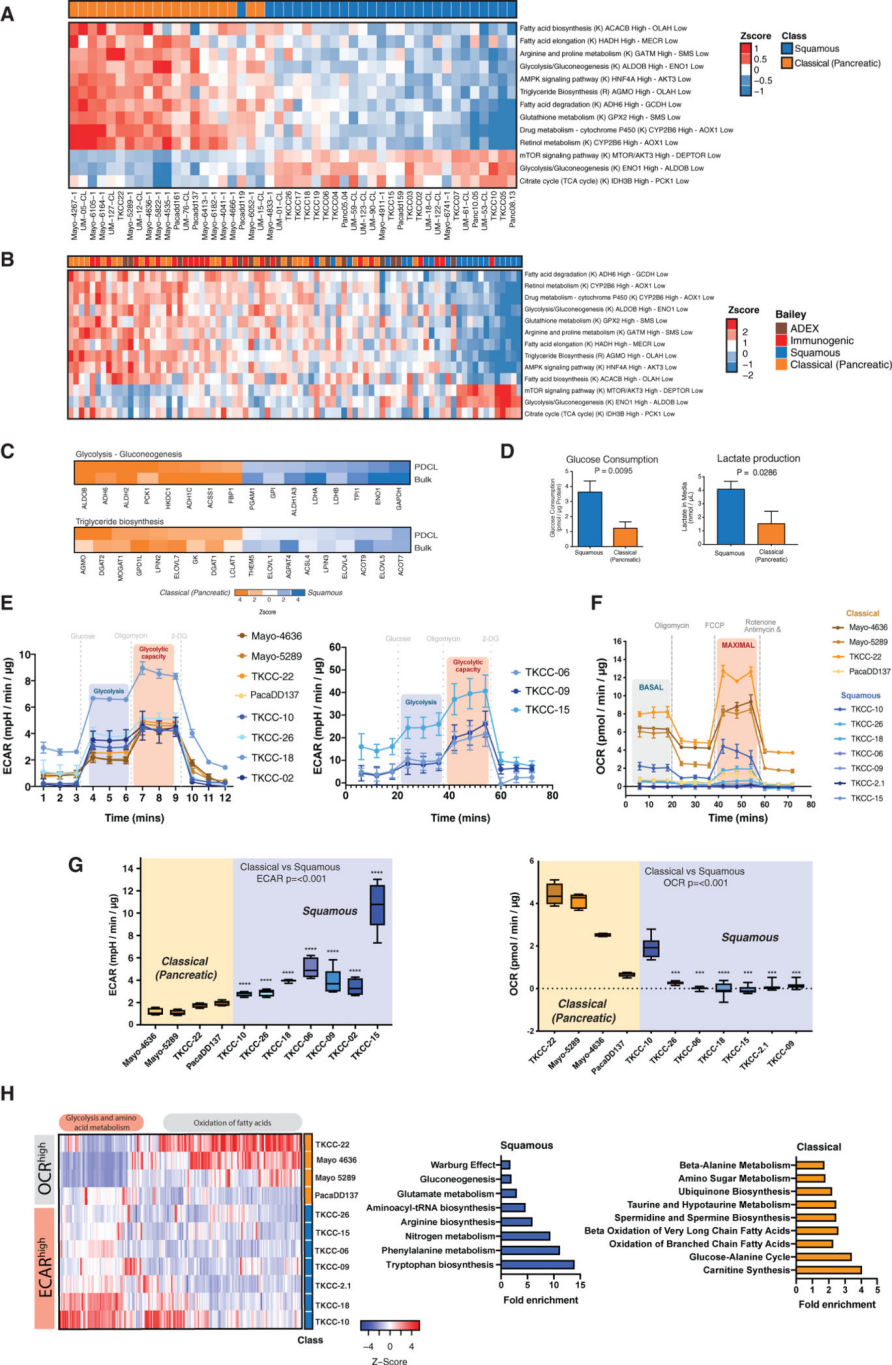
Metabolic Differences Between Squamous and Classical (Pancreatic) PDCLs (A) Heatmap of pathways and molecular processes involved in cancer metabolism showing enrichment of transcripts in pathways important in mTOR signaling and glycolysis in the squamous subtype. PDCLs were ranked from most classical (pancreatic) (orange) to most squamous (blue), using gene expression or pathway activity, and grouped into metabolic processes. PDCL ID is listed below the heatmap. (B) The same signature from (A) was applied to the RNA-seq cohort of bulk tumor from Bailey et al. (2016). Subtype classification is depicted by annotated colors on the top row. The immunogenic subtype has a transcriptional signature associated with immune infiltrate and shares transcriptional networks associated the classical (pancreatic) subtype (Bailey et al., 2016). ADEX, aberrantly differentiated endocrine eXocrine subtype defined by transcriptional networks important for pancreatic differentiation. (C) Heatmaps of key genes involved in glycolysis-gluconeogenesis and triglyceride biosynthesis. Genes are ranked by most differentially expressed between classical (pancreatic) (orange) and squamous (blue) subtypes of PDCLs, with color saturation proportional to degree of either classical or squamous enrichment, which is compared with and on the whole recapitulated in bulk tumors. (D) Relative lactate release and glucose consumption from squamous (TKCC-10 and TKCC-26) and classical (pancreatic) (TKCC-22, Mayo 5289, and Mayo 4636) PDCLs were determined by colorimetric analysis. Raw values were normalized to cell counts. (E) Glycolysis activity profile of squamous and classical (pancreatic) PDCLs using Agilent Seahorse XF Glycolysis Stress Test. (F) Agilent Seahorse XF Cell Mito Stress Test profiles of squamous and classical PDCLs. (G) Left: ECAR values for cells treated as in (E) corrected for non-glycolytic acidification. Right: OCR values for cells treated as in (F) corrected for oxygen consumption resultant from processes other than mitochondrial respiration. Boxplots are annotated using one-way ANOVA. Error bars represent mean ± SD. Independent experiments are shown, n = >6. ***p ≤ 0.001, ****p ≤ 0.0001. (H) Left: untargeted metabolomic analysis of indicated PDCLs. Right: metabolite pathway enrichment analysis of significantly altered metabolites between classical and squamous PDCLs. See also Figures S1 and S2 and Tables S1, S2, S3, and S4.

We have previously demonstrated that transcriptional networks involved in energy source generation differ substantially between the classical (pancreatic) and squamous subtypes (Bailey et al., 2016). Comparative analysis of bulk tumor and PDCL transcriptomes demonstrated that PDCLs faithfully recapitulate the two broad PDAC transcriptomic subtypes observed in bulk tumor samples (Figures S1A and S2A; Table S1). Importantly, several gene programs representing key metabolic processes were highly preserved in PDCLs and, in keeping with our previous analyses, exhibited subtype-specific enrichment (Figures 1A–1C; Table S1). Squamous PDCLs were enriched for transcripts regulating mammalian Target Of Rapamycin (mTOR) signaling and glycolysis, in particular *AKT3* and Enolase 1 (*ENO1*), respectively, whereas the classical (pancreatic) PDCLs were enriched for fatty acid biosynthesis and elongation processes, such as the gene encoding the rate-limiting enzyme in fatty acid biosynthesis acetyl-coenzyme A (CoA) carboxylase β (*ACACB*) and the beta-oxidation pathway enzyme hydroxyacyl-CoA dehydrogenase (*HADH*). Liquid chromatography-mass spectrometry (LC-MS) analysis supported these findings and revealed subtype-specific differences in metabolite pools, with an enrichment of glycolysis intermediates in squamous PDCLs (Figure 1H; Table S2). Similarly, squamous PDCLs were associated with increased extracellular acidification rates (ECARs; indicative of lactate accumulation) and decreased oxygen consumption compared with classical (pancreatic) PDCLs (Figures 1E–1G, and S1B; Table S3). Functional assessment of glucose uptake and lactate production further supported this analysis, with increased glucose uptake and lactate production indicative of increased glycolytic flux in squamous PDCLs (Figure 1D; Table S3). Collectively, these data suggest that squamous PDCLs are highly catabolic and utilize glycolysis as their main source of energy.

Glycolytic gene expression, glucose uptake, and lactate secretion are increased in homozygote *KRAS*^G12D/G12D^ mutated lung cancer cells relative to *KRAS*^G12D/WT^ heterozygous (Kerr et al., 2016); therefore, the difference in glycolytic activity between classical (pancreatic) and squamous PDCLs may be a consequence of difference in *KRAS* copy number. However, DNA sequencing analysis established that *KRAS*^G12D^ heterozygotes and homozygotes were present across both subtypes (Figure S1C; Table S4). Enhanced glycolysis is a well-established phenotype of cancer that is typically associated with increased growth demands and/or compensatory adaptation to mitochondrial defects (Lin et al., 2012; Vander Heiden et al., 2009). Mitochondrial gene mutations were similar across subtypes (Figure S1D), suggesting that mitochondrial mutations were not driving a switch toward glycolysis, and growth rates were not significantly different between subtypes (Figures S1E and S1F). These data suggest that either differential KRAS dependency (Singh et al., 2009) exists between classical (pancreatic) and squamous PDCLs, or a further genetic or epigenetic event is required to switch cells toward a squamous-like metabolic preference for glycolysis.

### Loss of HNF4A or GATA6 in Classical (Pancreatic) PDCLs Recapitulates Transcriptional Profiles Associated with the Squamous Subtype

**Figure 2.**
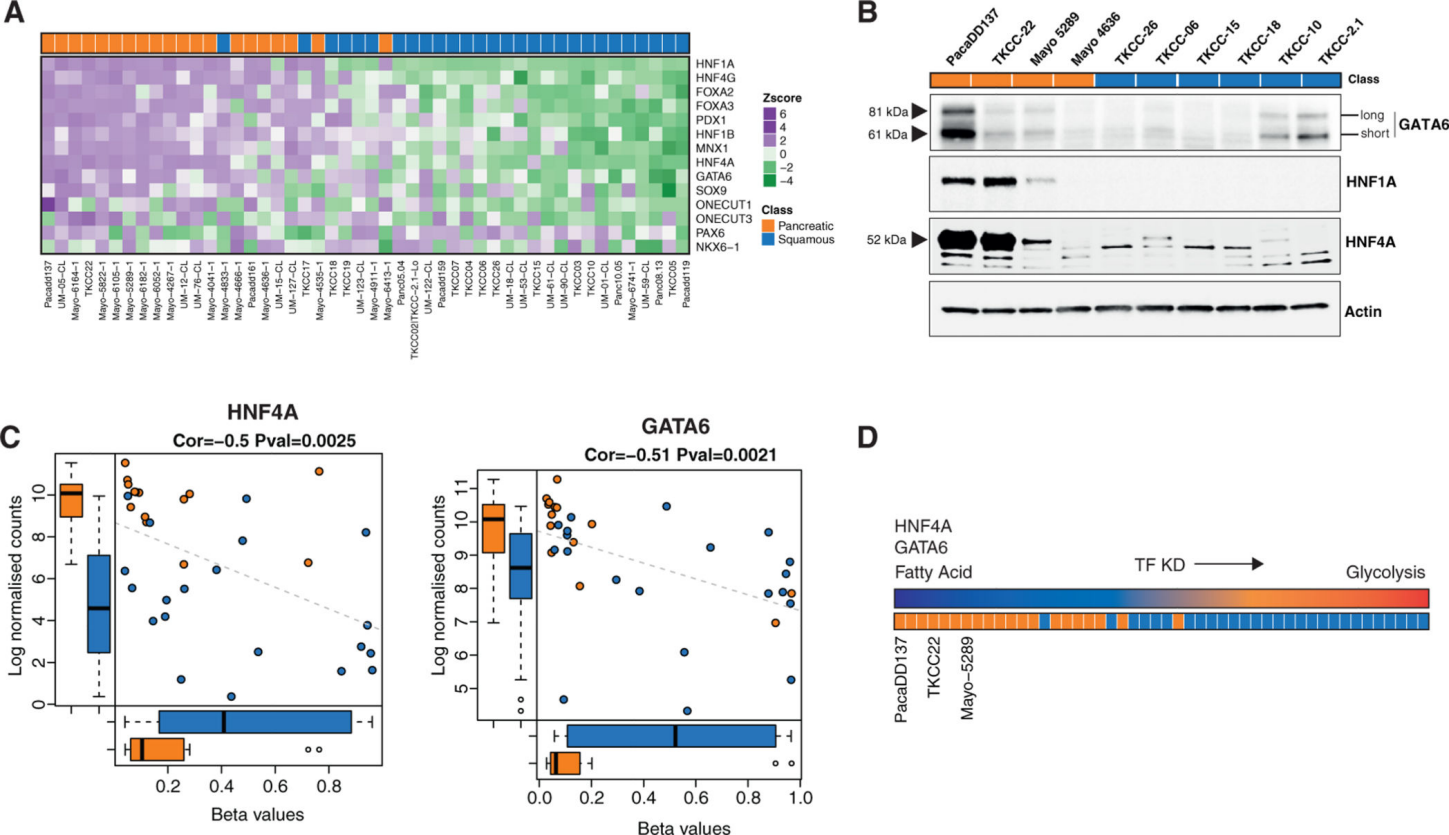
Subtype-Specific Differences in Endodermal TF Expression (A) Heatmap showing differential expression of regulatory genes central to pancreatic endodermal cell fate determination. Note loss of pancreatic transcripts *HNF4A* and *GATA6* in the squamous subtype indicated by RNA-seq analysis. (B) Immunoblots of endodermal cell fate determining transcription factors across a selection of PDCLs representative of both classical (pancreatic) and squamous subtypes. 20 μg of the same protein lysate was probed with stated antibodies on different blots. Actin panel is a representative loading control (HNF1A loading shown). (C) Plots showing regulation of gene expression by methylation. Methylation of *HNF4A* (left) or *GATA6* (right) is associated with the concordant downregulation of the indicated gene expression. Pearson correlation and adjusted p values are provided for each gene methylation comparison. Boxplot colors designate class: squamous (blue) and classical (pancreatic) orange. (D) Schematic representation of where the selected classical (pancreatic) PDCLs rank in terms of subtype. Expression of genes involved in endodermal cell fate was used to rank subtype. See also Figure S2 and Table S1.

**Figure 3.**
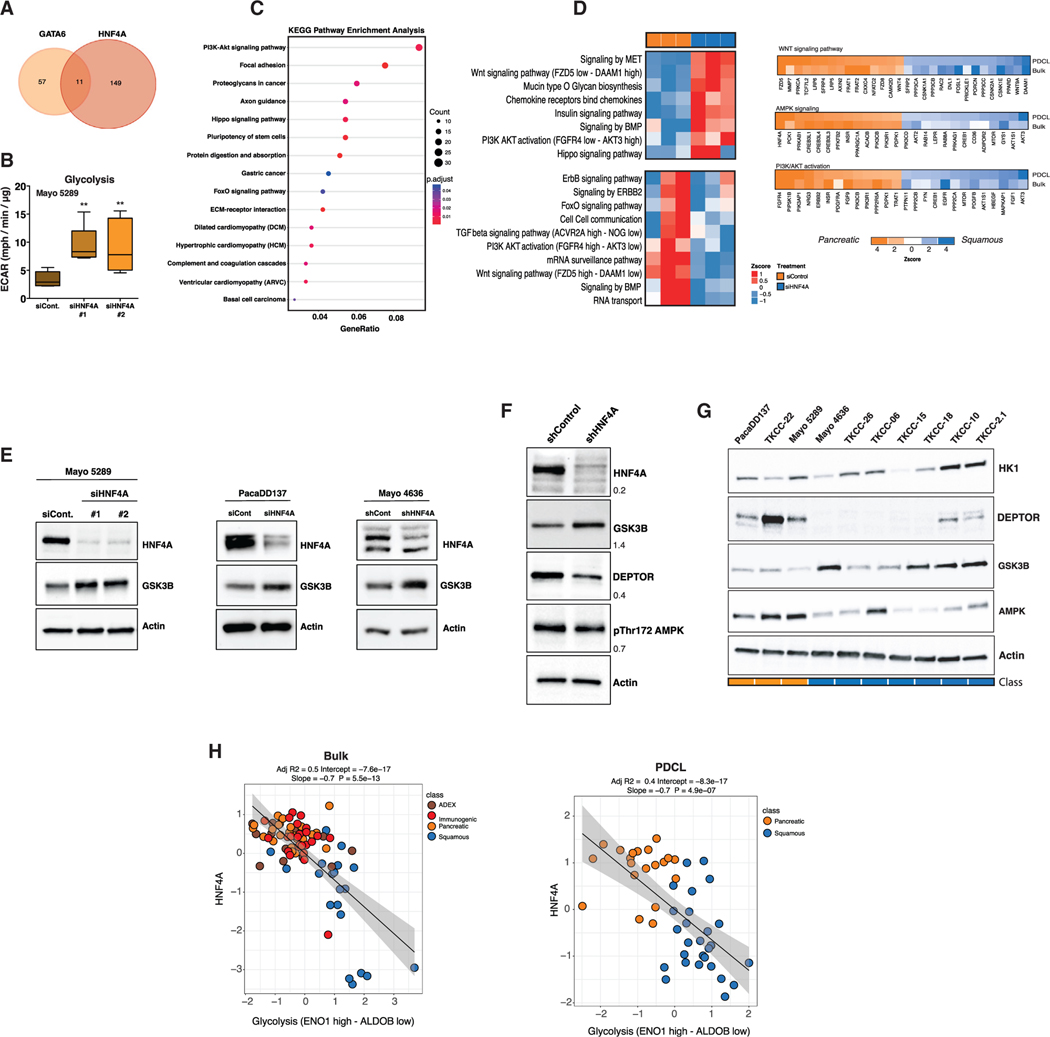
HNF4A Loss in Classical (Pancreatic) PDCLs Drives a Switch toward a Squamous-Associated Metabolic Profile (A) Venn diagram showing the number of common and unique genes differentially expressed (p ≥ 0.05, fold change ≥ 2) after either *HNF4A* or *GATA6* knockdown in the classical (pancreatic) Mayo 5289 PDCL. (B) ECAR in classical (pancreatic) PDCLs following siRNA-mediated knockdown of *HNF4A*. Boxplots are annotated using one-way ANOVA, mean ± SD. Technical replicates are shown, n ≥ 6. For all graphs: **p ≤ 0.01. (C) Kyoto Encyclopedia of Genes and Genomes (KEGG) pathway enrichment analysis of significantly altered pathways identified after *HNF4A* knockdown in Mayo 5289 PDCL. Adjusted p value for each annotation is represented by color scale. Gene ratio is represented by dot size. Enriched terms and pathways were identified as significant at an adjusted p value ≤ 0.05 and FDR ≤ 0.05. (D) Comparison of molecular pathways identified in bulk tumor and PDCLs RNA-seq analysis with significant gene changes following *HNF4A* knockdown. (E) Right: Mayo 5289 PDCLs treated with two independent *HNF4A* siRNA oligos for 72 h were immunoblotted with indicated antibodies. Left: transient or stable *HNF4A* knockdown in PacaDD137 and Mayo 4636 PDCLs, respectively. Actin panel is a representative loading control (HNF4A loading shown). (F) Stable *HNF4A* knockdown in Mayo 5289 PDCL immunoblotted with PI3K signaling proteins identified from RNA-seq analysis. Actin panel is a representative loading control (HNF4A loading shown). (G) A selection of PDCLs ranked from classical (pancreatic) to squamous immunoblotted with indicated antibodies. Actin panel is a representative loading control (DEPTOR loading shown). For all blots in (E)–(G), 20 mg of the same protein lysate was probed with stated antibodies on different blots. (H) Correlation graph demonstrating a negative correlation of HNF4A expression with glycolysis pathway expression from bulk tumor samples described by Bailey et al. (2016) (left) and in PDCLs (right). See also Figure S3 and Table S5.

We previously established that the squamous subtype is characterized by hypermethylation and concordant downregulation of genes that govern pancreatic endodermal cell-fate determination, such as *HNF1A*, *HNF4A*, and *GATA6*, leading to complete loss of endodermal identity (Bailey et al., 2016). Autosomal dominant mutations in *HNF4A* result in hereditary forms of diabetes mellitus referred to as maturity-onset diabetes of the young (MODY), which is characterized by metabolic reprogramming and early-onset, non-insulin-dependent diabetes that is closely related to pancreatic secretory dysfunction (Stride and Hattersley, 2002). Moreover, MODY patients have increased risk for developing pancreatic cancer (Ræder et al., 2014). Given that *HNF4A* and *GATA6* are frequently epigenetically silenced in squamous PDAC tumors (Bailey et al., 2016) and PDCLs (Figures 2A–2C), and mutations in these genes are associated with metabolic reprogramming, we tested whether loss of these transcription factors in a classical (pancreatic) genetic background would drive a switch toward glycolysis (Figure 2D). We focused our subsequent analysis on the Mayo 5289 PDCL because this cell line clearly separated into the classical (pancreatic) subtype following PCA analysis of RNA sequencing (RNA-seq) data (Figure S2B; Table S1) and expressed RNA and protein of each TF (Figures 2A and 2B). Using small interfering RNA (siRNA), we targeted *GATA6* or *HNF4A* in Mayo 5289 cells and performed RNA-seq analysis (Figures 3, S2C, and S2D; Table S5). As previously reported, we also observed that GATA6 suppresses the expression of a squamous-like molecular phenotype (Martinelli et al., 2017); in particular, gene set enrichment analysis (GSEA) revealed that loss of GATA6 in a progenitor genetic background led to dysregulation of gene programs involved in extracellular matrix organization and WNT ligand biogenesis and trafficking (Figure S2D). *HNF4A* knockdown was associated with increased ECARs indicative of increased glycolysis (Figure 3B; Table S5) and induced dysregulation of the phosphatidylinositol 3-kinase (PI3K)-AKT signaling pathway (Figures 3C and 3D). In particular, *HNF4A* knockdown was associated with a reduction in DEPTOR and an upregulation of WNT pathway signaling molecules WNT5A, WNT5B, WNT7B, and WNT10B (Figure 3D). When compared with the RNA-seq analysis of bulk tumors, HNF4A reduction recapitulated expression signatures associated with the squamous subtype, such as WNT and insulin signaling and PI3K-AKT activation (Figure 3D), suggesting that HNF4A loss drives metabolic reprogramming at an early stage of PDAC progression. To investigate the sufficiency of HNF4A loss to install squamous-like metabolic reprogramming, we further knocked down *HNF4A* in the classical (pancreatic) PDCLs PacaDD137, TKCC-22, and Mayo-4636 (Figure S3A; Table S5). *HNF4A* knockdown in the further subset of classical (pancreatic) PDCLs recapitulated our previous results and was associated with an increase in glycolysis.

### Loss of HNF4A Activates a Gene Expression Program that Favors Glycolysis

Rate-limiting enzymes that mediate glucose metabolism such as hexokinase I and II (*HK1* and *HK2*) were significantly induced in *HNF4A* knockdown PDCLs (Figures S3B and S3C). Increased expression of these enzymes is associated with the squamous subtype (Figure 3G). The gene encoding *ALDOB*, a glycolytic enzyme that catalyzes the conversion of fructose-1,6-bisphos-phate to glyceraldehyde-3-phosphate, decreased following HNF4A knockdown (Figure S3B), and a high ratio of *ALDOA* relative to *ALDOB* expression is associated with poor patient prognosis (Figure S3D). Furthermore, the AMP-activated protein kinase (AMPK) catalytic subunit *PRKAA1* was reduced following *HNF4A* knockdown, with low expression also associated with poor survival (Figures S3B and S3D). *GSK3B*, encoding a protein kinase that acts as a regulator of glucose homeostasis (Reya and Clevers, 2005) and WNT signaling (Wu and Pan, 2010), was also significantly increased following *HNF4A* knockdown, with higher protein expression also found to be associated with the squamous subtype (Figures 3E–3H). In classical (pancreatic) PDCLs with HNF4A knockdown, we consistently found increased ECAR (Figures 3B and S3A; Table S5) and increased *GSK3B* protein expression after *HNF4A* knockdown (Figures 3E and 3F). Collectively, these findings suggest that HNF4A loss can mediate a switch toward a squamous subtype metabolic profile and identify ALDOA, HK, and GSK3β as potential key molecular regulators of glycolysis in squamous PDAC.

### Targeting Glycolysis Shows Subtype Sensitivity in Squamous PDCLs

To corroborate these findings and identify key metabolic vulnerabilities that could be therapeutically targeted, we conducted an siRNA-mediated gene silencing screen of metabolic targets in a subset of PDCLs (Figure S4A; Table S6). Consistent with our previous findings, metabolic dependencies in squamous PDCLs were enriched for targets falling within glycolytic metabolic pathways (Figures S4A–S4C). Targeted inhibition of glycolysis using either glucose analog 2-deoxy-D-glucose (2-DG) or the pentose phosphate pathway (PPP) inhibitor 6-aminonicotinamide showed subtype-specific sensitivity in squamous PDCLs (Figure S4D).

**Figure 4.**
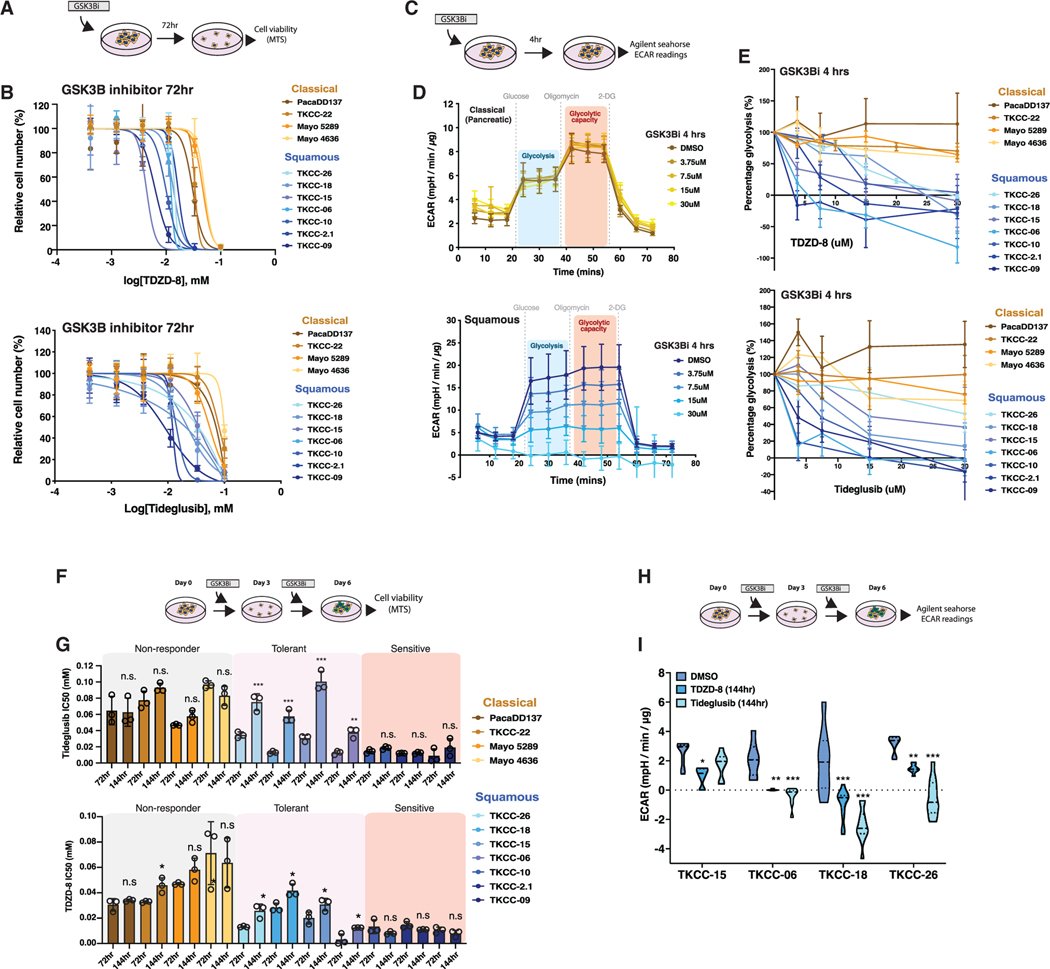
A Subset of Squamous PDCLs Acquires GSK3β Drug Tolerance after Chronic Suppression of Glycolysis (A and B) Schematic of experimental setup (A) and dose-response curves (mean ± SD) (B) for classical (pancreatic) and squamous PDCLs treated with TDZD-8 (GSK3βi) or tideglusib (GSK3βi) for 72 h. Independent experiments are shown, n ≥ 3. DMSO-treated cells were set to 100%. (C and D) Experimental setup (C) and (top) representative Glyco Stress Test curves for (D) classical (pancreatic) or (bottom) squamous PDCLs. (E) ECAR values (mean ± SD) after treatment with TDZD-8 or tideglusib for 4 h in classical (progenitor) and squamous PDCLs. Technical replicates are shown, n ≥ 5. (F and G) Schematic of experimental setup (F) and comparison of IC_50_ values (mean ± SD) (G) after either 72- or 144-h treatment with either TDZD-8 (GSK3βi) or tideglusib (GSK3βi) in PDCLs. Unpaired t test. Independent experiments are shown, n = 3. (H and I) Schematic of experimental setup (H) and ECAR values (I) after 144-h treatment with either TDZD-8 (GSK3βi) or tideglusib (GSK3βi) in GSK3βi-tolerant squamous (TKCC-15, TKCC06, TKCC-18, and TKCC-26) PDCLs. Technical replicates are shown, n = 8. For all graphs: *p < 0.05; **p % 0.01; ***p % 0.001; ****p < 0.0001. Figure legend colors designate class: classical (pancreatic) = orange/brown; squamous = blue. See also Figures S4 and S5 and Table S6.

With the objective of identifying therapeutically relevant targets, we selected GSK3β for further evaluation for the following reasons: (1) the previously established role for GSK3b in glucose homeostasis (Embi et al., 1980; Woodgett and Cohen, 1984), and (2) we consistently observed increased *GSK3B* expression and a concomitant induction of glycolysis following HNF4A knockdown. Furthermore, multiple phase 2 clinical trials (ClinicalTrials.org: NCT02586935, NCT01350362, NCT02858908) using GSK3β inhibitor tideglusib highlight the potential of this compound to effectively treat PDAC. As predicted, squamous PDCLs exhibited increased sensitivity to GSK3b inhibitors, TDZD-8 and tideglusib, in comparison with classical (pancreatic) PDCLs (Figures 4A, 4B, and S4D; Table S6), and importantly, glycolysis was selectively reduced in squamous PDCLs (Figures 4C–4E).

### A Subset of Squamous PDCLs Acquires GSK3β Drug Tolerance after Extended Suppression of Glycolysis

Recent reports have described adaptive metabolic networks that can compensate for metabolic targeting in PDAC (Biancur et al., 2017). To determine whether the anti-proliferative effects of GSK3β are sustainable after prolonged treatment, we extended our proliferation assays to 6 days. When comparing 72- and 144-h inhibitor incubations, we observed a significant increase in the half maximal inhibitory concentration (IC_50_) values for TDZD-8 and tideglusib in a subset of our squamous PDCLs (Figures 4F and 4G; Table S6), despite the sustained inhibition of glycolysis in these cells (Figures 4H and 4I). These data suggest that a subset of our squamous PDCLs can adapt to chronic suppression of glycolysis. We next sought to identify the molecular mechanism regulating metabolic adaptation in a subset of squamous PDCLs that enabled them to tolerate GSK3β inhibition.

GSK3β inhibition can modulate autophagy by increasing the LKB1-AMPK-ULK signaling pathway activity and induce drug tolerance (Sun et al., 2016). Recent studies have also shown that suppression of glycolysis via MAPK pathway inhibition in PDAC can lead to a greater dependency on autophagy, and that combinations targeting both MAPK signaling and autophagy synergistically suppress proliferation and induce apoptosis (Bryant et al., 2019; Kinsey et al., 2019). To determine whether autophagy was mediating GSK3β drug tolerance in this subset of squamous PDCLs, we tested the expression of known autophagy regulators AMPK and ULK after GSK3b inhibition. Indeed, we observed an increase in active phospho-AMPK (Thr172) and phosphor-ULK (Ser555) suggesting activation of autophagy after GSK3βi (Figure S5). However, combinatorial targeting of AMPK (Dite et al., 2018) and ULK (Egan et al., 2015) with SBI-0206965 and GSK3βi (TDZD-8 or tideglusib) resulted in only a modest rescue in inhibitor sensitivity and failed to rescue drug tolerance (Figure S5; Table S7). Only after high concentrations of SBI-0206965 (Figure S5) was toxicity observed, suggesting an alternative or additional mechanism for drug tolerance/resistance.

### ATAC-Seq and Transcriptomic Analysis Reveal a Uniquely Accessible WNT Gene Program in the Drug-Tolerant Squamous Subtype

In an effort to identify nodes of therapy resistance, we next sought to establish what key differences exist between groups of squamous PDCLs that show differential adaptation to GSK3β-mediated suppression of glycolysis. Recent studies have established that subtypes of PDAC are associated with distinct epigenetic landscapes (Andricovich et al., 2018; Bailey et al., 2016; Somerville et al., 2018), and that these chromatin states may underpin PDAC heterogeneity (Lomberk et al., 2018). Transcriptomic analysis of a human pancreatic tumor organoid library (PTOL) established that PDAC segregates into three subtypes with distinct methylation patterns and dependency on WNT niche signaling (Seino et al., 2018). Seino et al. (2018) showed that a subgroup of PDAC organoids designated as W+ had the ability to harness self-produced WNT ligands. GSK3β plays a central role in the regulation of the WNT/β-catenin signaling pathway. When the WNT ligand is present, it binds to specific membrane-bound receptors. This binding in turn activates an intracellular signaling cascade, which ultimately results in β-catenin stabilization and nuclear localization. In the nucleus, β-catenin associates with members of the TCF/LEF family of transcription factors to regulate the transcription of various WNT targets. GSK3β phosphorylates β-catenin triggering its degradation and consequently reducing β-catenin nuclear accumulation (Reya and Clevers, 2005; Wu and Pan, 2010).

Given the established function of GSK3β as a negative regulator of WNT-mediated β-catenin signaling (Aberle et al., 1997; He et al., 1995; Huang et al., 2017), we hypothesized that GSK3β inhibition may mimic WNT signaling through the direct stabilization of β-catenin, providing a survival advantage in a subset of cells capable of harnessing self-produced WNT ligands. We further reasoned that different chromatin landscapes could exist between subtypes of squamous PDCLs that would be predictive of those expected to attain drug tolerance and may explain the observed heterogenous response to targeted therapy. To address these questions, we performed an integrative analysis of ATAC-seq and RNA-seq data from our PDCLs.

**Figure 5.**
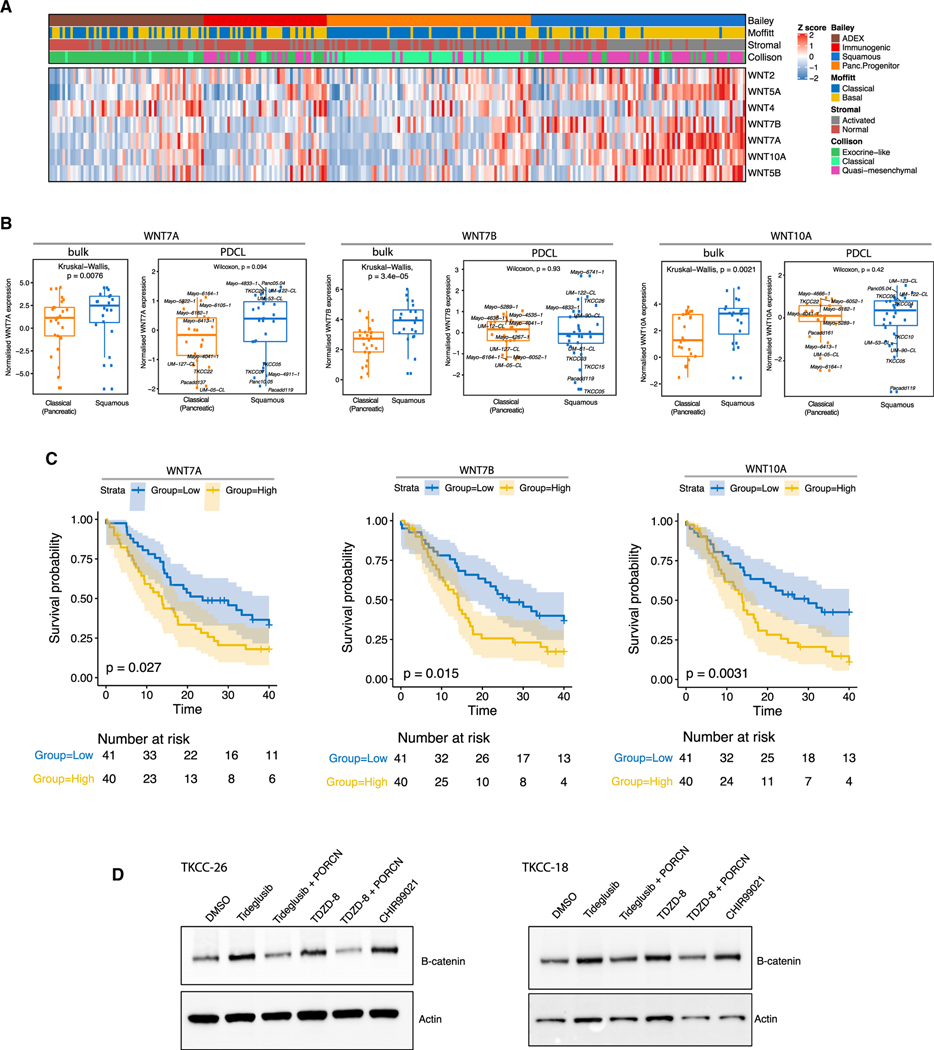
PDAC PDCLs Express WNT Ligands (A) Heatmap showing mRNA expression of indicated WNT ligands in PDAC subtypes determined by RNA-seq analysis. (B) Left: boxplots showing a significant association of *WNT7A*, *WNT7B*, and *WNT10A* expression in the squamous subtype from RNA-seq analysis of bulk tumor samples from Bailey et al. (2016). Kruskal-Wallis test. Right: boxplots showing WNT expression in the PDCLs. Wilcoxon test. (c) Kaplan-Meier plots showing overall survival based on data reported by Bailey et al. (2016). Tumor samples were stratified based on *WNT7A* (left), *WNT7B* (center), or *WNT10A* (right) expression. Blue shading represents patients with low *WNT7A*, *WNT7B*, or *WNT10A* expression, respectively. Yellow shading represents patients with high *WNT7A*, *WNT7B*, or *WNT10A* expression, respectively. Log rank p value. (D) Western blot for indicated targets in squamous PDCLs TKCC-26 and TKCC-18 after 24 h GSK3βi (tideglusib or TDZD-8) ± PORCN (LGK-974). GSK3α/β (CHIR99021) was used as a positive control. See also Figure S6.

We first established whether our PDCLs and PDAC subtypes expressed WNT ligands. Consistent with previous reports in PDAC organoids (Seino et al., 2018), *WNT5A*, *WNT7A*, *WNT7B*, and *WNT10A* mRNA were highly expressed in PDCLs, suggesting a tumor cell-intrinsic origin for these WNT ligands (Figure 5A; Table S1). Furthermore, high expression of *WNT7A*, *WNT7B*, and *WNT10A* in clinical PDAC samples (Bailey et al., 2016) was associated with poor survival (Figures 5B and 5C). We next tested β-catenin protein stabilization after GSK3β inhibition and as predicted found an increase in β-catenin protein expression (Figure 5D). Importantly, treatment with the porcupine inhibitor LGK-974 was able to reduce GSK3βi (TDZD-8 and tideglusib)-mediated β-catenin stabilization, suggesting that secretory WNT ligands are required to mediate this transcriptional effect (Figure 5D). These results demonstrate that a subset of PDAC PDCLs can autonomously activate WNT signaling by expressing epithelial WNT ligands, which are also predictive of clinical outcome.

**Figure 6.**
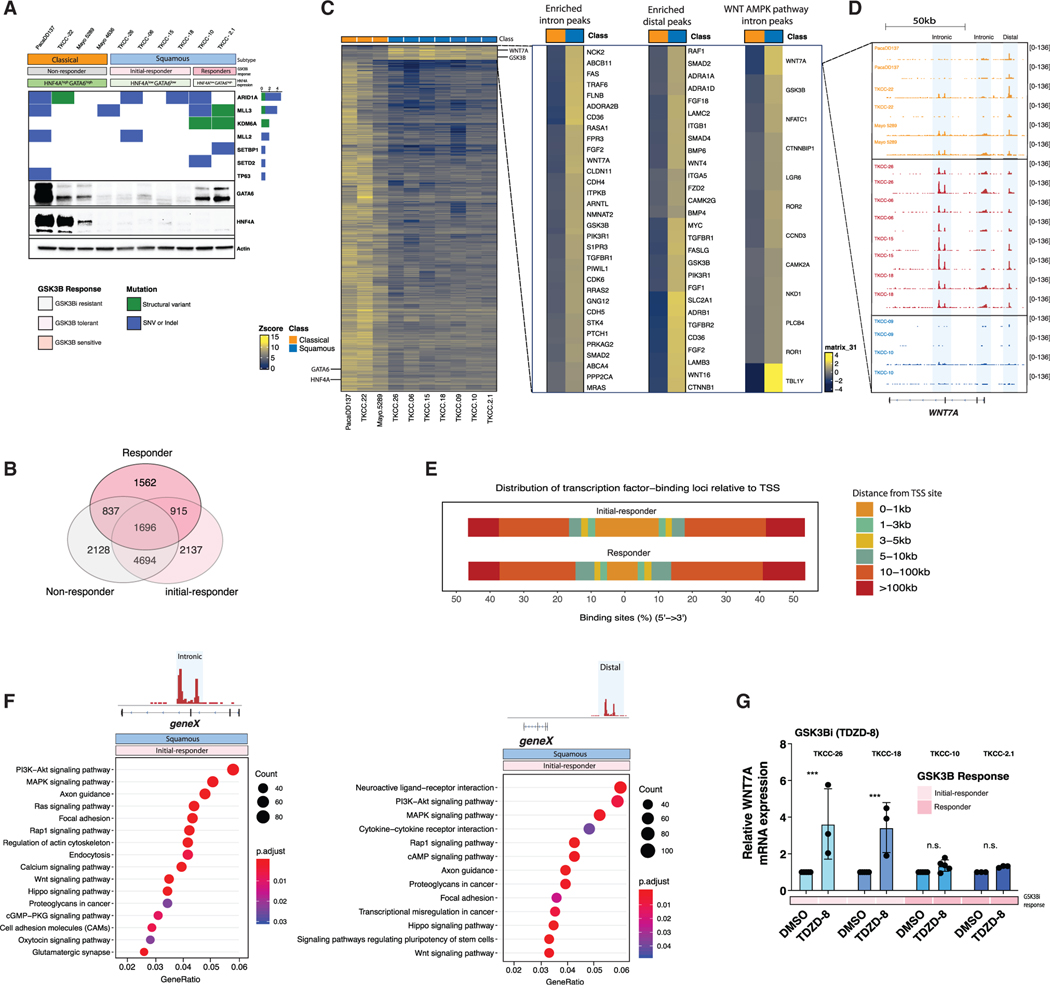
ATAC-Seq and Transcriptomic Analysis Revealed a Uniquely Accessible WNT Gene Program in Squamous PDCLs that Are Tolerant to GSK3B Inhibition (A) Western blot (WB) for either HNF4A or GATA6 in representative PDCLs of the classical (pancreatic) or squamous subtype. 20 μg of the same protein lysate was probed with stated antibodies on different blots. Actin panel is a representative loading control (HNF4A loading shown). (Above) Oncoplot showing somatic mutations in genes involved in chromatin regulation. Green = structural variant (SV); purple = single-nucleotide variant (SNV) or indel. (B) Venn diagram showing the number of common and unique annotated gene peaks in PDCLs grouped by response to GSK3βi. GSK3βi resistant = PacaDD137, TKCC-22, Mayo 5289; GSK3βi tolerant = TKCC-26, TKCC-06, TKCC-15, TKCC-18; GSK3βi sensitive = TKCC-09, TKCC-10, TKCC-2.1. (C) ATAC-seq density plots of accessible genes in 10 PDCLs representative of the classical (pancreatic) or squamous subtypes. (D) ATAC-seq genomic tracks for *WNT7A*. Highlighted regions show subtype-specific genomic peaks. PDCLs are grouped based on response to GSK3β inhibitor. (E) Chart showing the genomic distribution of ATAC-seq peaks in squamous PDCLs that are sub-grouped based on response to GSK3βi. (F) KEGG pathway enrichment analysis of enriched pathways accessible in GSK3β-tolerant squamous PDCLs found at intronic and distal promoter sites. (G) WNT7A expression in squamous PDCLs treated with GSK3b (TDZD-8) for 144 h. For all graphs: **p < 0.01; ***p < 0.0001. See also Figures S6 and S7 and Table S7.

With metabolic adaptation occurring only in a subset of squamous PDCLs, we next explored whether further classification based on GSK3β inhibitor response and chromatin accessibility could identify responsive subgroups. To this end, we ranked our PDCLs into three response groups: GSK3β non-responders (PacaDD137, TKCC-22, Mayo 5289, and Mayo 4636), GSK3β initial responders (TKCC-26, TKCC-06, TKCC-15, and TKCC-18), and GSK3β responders (TKCC-10, TKCC-2.1, and TKCC-09) (Figure 6A). Differential peak analysis of ATAC-seq was then performed to identify chromatin accessibility regions exhibiting significant change among the three GSK3βi response groups (Figures 6B and 6C). Loss of chromatin accessibility proximal to *HNF4A* and *GATA6* gene loci was associated with a concomitant increase in chromatin accessibility proximal to the WNT7A and GSK3β gene loci (Figures 6C and 6D; Table S7). Direct comparison of chromatin accessibility at the *WNT7A* locus revealed that the subset of squamous PDCLs that demonstrated acquired resistance to GSK3β inhibition was enriched for both intronic and distal promoter peaks (TKCC18, TKCC-06, TKCC-15, and TKCC-26); however, loss of these peaks was observed in the GSK3βi-sensitive subgroup (TKCC-10, TKCC-2.1, and TKCC-09) (Figures 6C and 6D). In line with reports that squamous PDAC subtypes rely on super-enhancers to mediate transcription in a highly methylated chromatin landscape (Lomberk et al., 2018; Somerville et al., 2018), we observed that chromatin accessibility peaks in the GSK3βi-sensitive subgroup were enriched within distal elements, suggesting a role for super-enhancers in regulating gene expression in this subset of cells (Figure 6E; Table S7). GSEA using intronic and distal peaks revealed that GSK3βi-tolerant squamous PDCLs exhibit increased chromatin accessibility in subsets of genes associated with WNT, PI3K-AKT, and Hippo signaling (Figures 6F and S6A–S6F). WNT7A was a significant hit in this analysis (Figures S6B and S6C). Collectively, these data suggest that GSK3βi-tolerant squamous PDCLs have access to an amplified WNT signaling program via application of both intronic and distal promoter usage, which may contribute to the acquired resistance to GSK3β inhibition observed in a subgroup of the squamous PDCLs. We next tested our GSK3βi-tolerant (TKCC-26 and TKCC-18) and -sensitive (TKCC-10 and TKCC-2.1) squamous PDCLs with extended GSK3βi treatment, and as predicted by the observed enrichment of chromatin accessibility in these cells, WNT7A expression increased in the GSK3βi-tolerant subgroup, but not the GSK3βi-sensitive subgroup (Figure 6G).

To identify putative transcriptional regulators enriched in regions of differential chromatin accessibility, we performed transcription factor motif analysis using HOMER (Heinz et al., 2010). Consistent with our RNA-seq analysis and reports in low-grade (Lo-G) PDAC (Diaferia et al., 2016), the GSK3βi-resistant subgroup, which is composed of classical (pancreatic) PDCLs, was enriched for TF motifs involved in endocrine specification, such as HNF6, HNF4A, and HNF1A (Figure S7A). The GSK3βi-sensitive subgroup was enriched for Activating Enhancer-Binding Protein 2 Gamma (AP-2 gamma) binding motifs. AP-2 is a transcription factor that facilitates the opening of distal enhancer regions (Pastor et al., 2018) (Figure S7A), further supporting the notion that squamous PDCLs rely on super-enhancers to mediate transcription. We next established which TF motifs were enriched in the GSK3β drug-tolerant subgroup (Figure S7A) with the further objective of identifying potential TFs that regulate WNT expression. Using orthogonal measures of motif enrichment, we identified RNA and protein expression Activating Transcription Factor-3 (ATF-3) (Figures S7A–S7D; Table S7) as a putative regulator of WNT gene expression in PDAC. ATF-3 has previously been established as a regulator of WNT ligand expression (Yan et al., 2011), suggesting ATF-3 as a potential candidate for *WNT7A* regulation in the GSK3βi-tolerant subgroup. Collectively, these data demonstrate that chromatin accessibility can be used to stratify squamous PDAC PDCLs into two subgroups that have differential access to TF binding motifs.

### Porcupine Inhibition Overcomes WNT-Driven Acquired Resistance to GSK3β Inhibition in Squamous PDCLs

**Figure 7.**
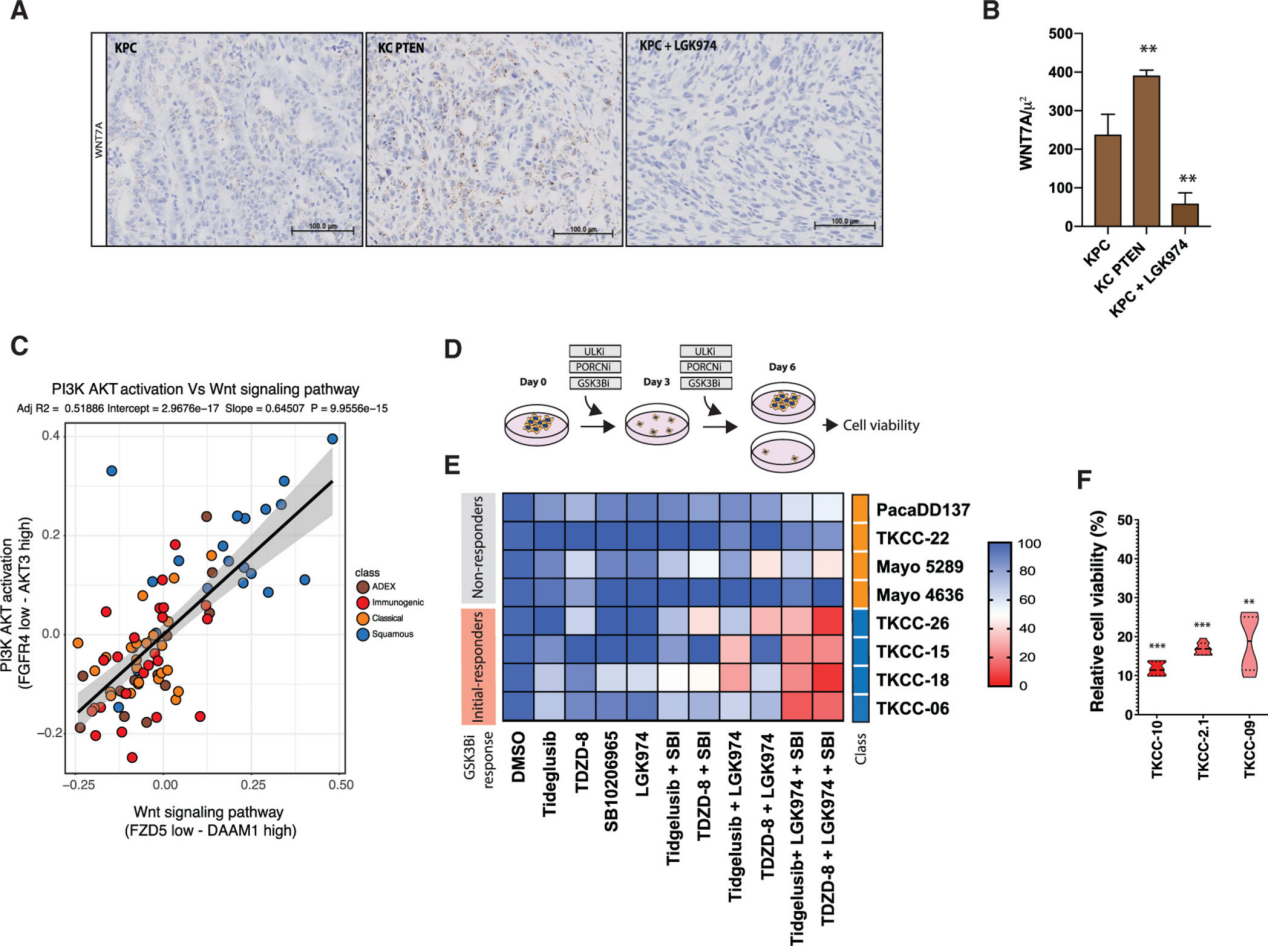
Porcupine Inhibition Overcomes WNT-Driven Acquired Resistance to GSK3β Inhibition (A) RNAscope hybridization for Wnt7a in PDAC GEMM KPC, KC Ptenfl/+, and KPC + LGK974 (porcupine inhibitor). Nuclear counterstaining is with hematoxylin. The scale bar represents 100 μm. (B) Quantification of samples described in (A) using HALO software. (C) Correlation graph demonstrating a positive correlation of PI3K-AKT activation with WNT signaling in the squamous subtype from bulk tumor samples described by Bailey et al. (2016). (D) Schematic of experimental setup. (E) Indicated PDCLs treated with either GSK3βi (tideglusib or TDZD-8), AMPKi/ULKi (SBI), or Porcupine-I (LGK974) alone or in combination for 144 h before cell number analysis. (F) GSK3β-sensitive squamous PDCLs (TKCC-10, TKCC-2.1, and TKCC-09) were treated with GSK3β(TDZD-8) for 144 h. Note that these cells remain sensitive to GSK3β(TDZD-8)-targeted therapy. **p ≤ 0.01; ***p ≤ 0.001. See also Table S7.

To determine whether dysregulation of PI3K signaling is associated with increased WNT expression, we utilized a previously described GEMM of pancreatic cancer harboring an oncogenic *Kras* mutation and deletion of *Pten* (KCPTEN) (Kennedy et al., 2011; Morran et al., 2014). RNAscope analysis of *Wnt7a* revealed that, similar to HNF4A/GATA6 loss in squamous PDCL (Figure 7B), an increase in PI3K signaling via phosphatase and tensin homolog (PTEN) loss was associated with higher expression of *Wnt7a*, and importantly, treatment with the porcupine inhibitor LGK-974 was able to reduce *Wnt7a* expression (Figures 7A and 7B; Table S7). These results demonstrate that activation of the PI3K pathway is associated with an increase in WNT7A expression, which can be suppressed by porcupine inhibition.

Having established that PDAC PDCLs can harness their own WNT-mediated β-catenin signaling, and that GSK3β inhibition amplifies this signaling in a subset of squamous PDCLs, we next determined whether porcupine inhibitors could effectively suppress WNT signaling in combination with GSK3β- and AMPK-targeted therapy. In squamous PDCLs that had previously tolerated long-term GSK3β inhibition, porcupine inhibition sensitized cells to GSK3β and ULK inhibition (Figures 7D and 7E). Combination treatment resulted in a reduction of cell proliferation and induced cytotoxicity (Figures 7D–7F; Table S7).

## DISCUSSION

Prior studies have shown that PDAC is composed of two broad transcriptomic subtypes, and that these subtypes are characterized by unique chromatin landscapes (Bailey et al., 2016; Collisson et al., 2019). We show that chromatin accessibility is an important and largely undescribed biomarker for the delineation of therapeutic subtypes that are otherwise indistinguishable by transcriptomic analysis.

Due to the lack of defined genetic mutations or biomarkers in PDAC that are predictive of therapeutic response to targeted therapies, and the observed differential response to glycolysis inhibition with metabolic adaptation in a subset of squamous PDCLs, we reasoned that stratification of PDAC using chromatin accessibility maps and transcriptomic data represents a method to identify patients who would respond to therapies targeting metabolism. ATAC-seq identified amplified WNT signaling via intronic and distal promoter usage in a subset of the squamous PDCLs. Importantly, this analysis and other recent studies demonstrate that the squamous subtype can be stratified into additional subgroups that may inform response to therapy (Chan-Seng-Yue et al., 2020). Accordingly, deeper analysis of chromatin accessibility profiles may reveal further therapeutically relevant subgroups in PDAC. A chromatin-mediated drug-tolerant state in cancer subpopulations has previously been described where inhibition of HDAC activity prevented the development of drug resistance. The histone demethylase KDM5A was found to be required for drug tolerance, suggesting that mutations in chromatin-modifying complexes would be expected to reduce plasticity. Indeed, the TKCC-10 and TKCC-2.1 PDCLs, which remained sensitive to targeted therapy, have a high chromatin modifier mutational burden (Table S4). The chromatin modifier KDM6A, which has been implicated in the progression of squamous PDAC (Andricovich et al., 2018), is a common mutation shared by GSK3b inhibitor-sensitive PDCLs, and in keeping with reported findings, we observe squamous-like pancreatic cancer in these PDCLs despite the presence of GATA6.

Recent evidence also demonstrates that novel GSK-3 inhibitor 9-ING-41, which is currently being evaluated in a phase I/II trial in patients with advanced cancer, can inhibit the growth of PDAC cells *in vitro* and xenografts *in vivo*. Importantly, 9-ING-41 sensitizes PDAC cells to gemcitabine by short-circuiting the ATR/Chk1 DNA damage response signaling pathway, providing a rationale for treatment regimens comprising specific GSK3 inhibitors in combination with standard-of-care chemotherapies such as gemcitabine and Abraxane (Ding et al., 2019). In addition, early results from the COMPASS trial suggest that first-line chemotherapy is associated with significantly better outcomes in patients with tumors falling within the classical PDAC RNA subtype (Aung et al., 2018). Based on these findings, optimum strategies for GSK3B stand-alone and/or combination therapies should include an assessment of PDAC RNA subtype and/or PDAC chromatin accessibility.

We demonstrated that plasticity exists between subtypes, and that siRNA-mediated loss of HNF4A and GATA6 can drive reprogramming from a classical (pancreatic) to predominantly squamous-associated transcriptional signature. The squamous subtype is associated with high mutational burden and a multitude of chromosomal rearrangements (Bailey et al., 2016); therefore, reverting this subtype back to a progenitor-associated phenotype would be expected to be more challenging than promoting a switch from classical (pancreatic) to squamous. However, under certain circumstances, reprogramming from a predominantly squamous to classical (pancreatic) subtype has been observed; for example, targeted ablation of myeloid cells in KPC GEMMs by the selective inhibition of CSF1R produces a profound shift in subtype (Candido et al., 2018). These data highlight an important paracrine role for the stroma in pancreatic cancer (PC). Likewise, stromal cues have been shown to drive distinct changes in tumor cell metabolic pathways and to re-program the tumor epigenome (Sherman et al., 2017). Whether a stroma contribution to therapy-sensitive PDCLs (TKCC-10 and TKCC-2.1) would induce drug tolerance is yet to be determined.

Establishing whether a persisting subpopulation of PDAC cells contributes to resistance to targeted therapy or whether dynamic fluctuations of protein expression at the single-cell level explain the development of therapeutic resistance remains unanswered. Future studies will be directed at understanding how therapy-induced tumor evolution or cell population selection evolves at the single-cell level, and how enhancer and chromatin reprogramming participate in mediating drug tolerance. Identifying key regulators of these switching events could ultimately prevent therapy-induced tumor evolution. Predicted targets are expected to be directed toward chromatin remodelers and transcriptional enhancers.

A patient selection strategy based on chromatin profiling could identify patients for GSK3β-targeted therapy. The squamous PDCLs that remained sensitive to GSK3β inhibition have mutations in LRP6 (TKCC-2.1), LKB1 (TKCC-10), and chromatin modifiers KDM6A, ARID1A, SETD2, SETBP1, and MLL3 (Table S6). LRP6 is a receptor that transduces WNT-mediated signaling through the canonical WNT pathway (Garg et al., 2017), and LKB1 is a protein kinase responsible for activating AMPK (Shackelford and Shaw, 2009). This suggests that both functional WNT and AMPK signaling are required to mediate GSK3β inhibitor tolerance; therefore, patients identified as squamous, with a chromatin profile that promotes distal promoter usage, possibly KDM6A mutant, and harboring either LRP6 or LKB1 mutations would be predicted to maintain sensitivity to GSK3β-targeted monotherapy.

## STAR★METHODS

### LEAD CONTACT AND MATERIALS AVAILABILITY

Further information and requests for resources and reagent should be directed to and will be fulfilled by the Lead Contact, Dr Peter Bailey. Distribution of Mayo and PacaDD PDCLs are restricted by Material Transfer Agreements (MTAs). TKCC PDCLs are available upon request from the Australian Pancreatic Cancer Genome Initiative (APGI) at https://www.pancreaticcancer.net.au/bioresource-pdcls/.

### EXPERIMENTAL MODEL AND SUBJECT DETAILS

#### Human Subjects

APGI: Sydney South West Area Health Service Human Research Ethics Committee, western zone (protocol number 2006/54); Sydney Local Health District Human Research Ethics Committee (X11– 0220); Northern Sydney Central Coast Health Harbour Human Research Ethics Committee (0612– 251M); Royal Adelaide Hospital Human Research Ethics Committee (091107a); Metro South Human Research Ethics Committee (09/ QPAH/220); South Metropolitan Area Health Service Human Research Ethics Committee (09/324); Southern Adelaide Health Service/Flinders University Human Research Ethics Committee (167/10); Sydney West Area Health Service Human Research Ethics Committee (Westmead campus) (HREC2002/3/4.19); The University of Queensland Medical Research Ethics Committee (2009000745); Greenslopes Private Hospital Ethics Committee (09/34); North Shore Private Hospital Ethics Committee. Johns Hopkins Medical Institutions: Johns Hopkins Medicine Institutional Review Board (NA00026689). Ethik-kommission an der Technischen Universität Dresden (Approval numbers EK30412207 and EK357112012). University of Michigan Institutional Review Board (HUM00025339). Mayo Clinic Institutional Review Board (# 66–06)

#### Cell Lines

Patient derived cell lines (PDCLs) were generated as previously described (Chou et al., 2018; Pal et al., 2014; Rückert et al., 2012; Waddell et al., 2015). PDCLs were cultured in conditions specifically formulated for each individual line based on growth preferences and those resulting in cell lines that most closely resembled physiological cells from the initial tumor. Detailed culture media formulations for TKCC PDCLs are previously described in Hardie et al. (2017). Mayo PDCLs were cultured in DMEM/F12 (Life technologies, #11320–074) supplemented with 10% FBS (ThermoFisher Scientific, #SH30084.03) and 15mM HEPES (Life technologies, #15630–049). PacaDD lines were grown in DMEM (Life technologies, #41965–039), 10% FBS and KSFM formulation (Life technologies, #17005–059, Life technologies, #37000–015). Cells were grown in a humidified environment with either 5% or 2% O_2_. All cell lines were profiled by short tandem repeat (STR) DNA profiling as unique (CellBankaustralia.com). Cell lines were tested routinely for mycoplasma contamination using MycoAlert PLUS Mycoplasma Detection Kit (Lonza, #LT07 – 318). Information on the sex of the PDCLs is not available. HEK293T cells were obtained from the American Type Culture Collection (ATCC CRL-11268) and maintained in DMEM (Life Technologies, #11960044) supplemented with 10% FBS and 2mM L-glutamine (Life Technologies, #25030081).

#### *In vivo* animal studies

Pdx1-Cre, LSL-Kras^G12D^, Ptenfl, and LSL-Trp53R172H mice have been described previously (Hingorani et al., 2005; Kennedy et al., 2011). Mice on a mixed strain background were kept in conventional animal facilities and experiments carried out in compliance with UK Home Office guidelines and approved by the University of Glasgow Animal Welfare and Ethical Review Board. Mice were genotyped by Transnetyx (Cordova, Tennessee, USA). Adult mice of both sexes were used in studies. Mice were treated with 5mg/kg LGK974 in 0.5% methylcellulose / 0.5% Tween 80, p.o. BID. Animals were sacrificed as per institutional guidelines, and tissues removed and fixed in 10% buffered formalin.

### METHOD DETAILS

#### Western blotting

Protein lysates were harvested in RIPA lysis buffer supplemented with PhosSTOP easypack (Roche, #04906845001) and cOmplete Protease Inhibitor Cocktail (Roche, #4693116001) and quantified using Pierce BCA protein assay kit (ThermoFisher, #23225). Following SDS-PAGE, proteins were transferred to Nitrocellulose membranes (Amersham Biosciences, #45–001-227). To block, membranes were incubated in Tris-buffered saline (TBS) containing 5% BSA (Sigma, #A7906) and 0.1% Tween 20 (TBS-T) for 1hr before incubation with the primary antibody overnight at 4°. Membranes were then washed with TBS-T followed by incubation with secondary antibodies (Anti-Mouse IgG, Jackson ImmunoResearch #715–035-150, anti-Rabbit IgG Jackson ImmunoResearch #111–035-144) for 1hr at room temperature. Membranes were visualized using Pierce ECL western blotting substrate (ThermoFisher Scientific, Cat #32106) on BioRad chemiDoc MP Imaging system. Antibodies used are listed in STAR Methods Key Resources Table.

#### Nucleic acid extraction

DNA and RNA extractions were performed using QIAGEN DNeasy Blood & Tissue kit (Cat #69504) or QIAGEN RNeasy Mini kit (Cat #74104) respectively, according to manufacturer’s specifications.

#### Quantitative RT-PCR

cDNA was synthesized according to AffinityScript Multiple temperature cDNA synthesis kit instructional manual (Agilent Technologies, Cat #200436). Quantitative reverse transcription (RT)-PCR analyses were performed using SYBR Select Master Mix (ThermoFisher, Cat #4472903) according to reference manual and signals were acquired using QuantStudio 3 (ThermoFisher Scientific). GAPDH mRNA levels were used for data normalization. Each experiment was performed in triplicate. The primers used for quantitative RT-PCR are listed in the Key Resources Table.

#### Whole-genome library preparation

Whole-genome libraries were generated using either the Illumina TruSeq DNA LT sample preparation kit (Illumina, Part no. FC-121–2001 and FC-121–2001) or the Illumina TruSeq DNA PCR-free LT sample preparation kit (Illumina, Part no. FC-121–3001 and FC-121–3002) according to the manufacturer’s protocols with some modifications (Illumina, Part no. 15026486 Rev. C July 2012 and 15036187 Rev. A January 2013 for the two different kits respectively). For the TruSeq DNA LT sample preparation kit, 1 μg of gDNA was used as input for fragmentation to ~300 bp, followed by a SPRI-bead clean up using the AxyPrep Mag PCR Clean-Up kit (Corning, Part no. MAG-PCR-CL-250). After end-repair, 3ʹ adenylation and adaptor ligation, the libraries were size-selected using a double SPRI-bead method to obtain libraries with an insert size ~300 bp. The size-selected libraries were subjected to 8 cycles of PCR to produce the final whole-genome libraries ready for sequencing. For the TruSeq DNA PCR- free LT sample preparation kit, 1 μg of gDNA was used as input for fragmentation to ~350 bp, followed by an end-repair step and then a size-selection using the double SPRI-bead method to obtain libraries with an insert size ~350 bp. The size-selected libraries then underwent 3ʹ adenylation and adaptor ligation to produce final whole genome libraries ready for sequencing. Prior to sequencing, whole-genome libraries were qualified via the Agilent BioAnalyzer 2100 with the High Sensitivity DNA Kit (Agilent, Part no. 5067–4626). Quantification of libraries for clustering was performed using the KAPA Library Quantification Kit - Illumina/ Universal (KAPA Biosystems, Part no. KK4824) in combination with the Life Technologies Viia 7 real time PCR instrument.

#### RNA sequencing library generation and sequencing

RNA-seq libraries were generated as described in TruSeq Stranded Total RNA Sample Preparation Guide (illumina, part no. 15031048 Rev. E October 2013) using Illumina TruSeq Stranded Total RNA LT sample preparation kit. Ribosomal depletion step was performed on 500 ng of total RNA using Ribo-Zero Gold (Illumina, 20020598 and 20020492) followed by a 8 min heat fragmentation step aimed at producing libraries with an insert size between 120bp-200bp. First strand cDNA was synthesized from the enriched and fragmented RNA using SuperScript II Reverse Transcriptase (Thermofisher, 18064014) and random primers. Second strand synthesis was performed in the presence of dUTP. Following 3′ adenylation and ligation of adaptors to the dsDNA, libraries were subjected to 13 cycles of PCR. RNA-seq libraries were quantified using PicoGreen assay (Thermofisher, P11496) and sized and qualified using an Agilent 4200 TapeStation with Agilent D1000/High sensitivity ScreenTape (Agilent, 5067–5584). Libraries were normalized to 4nM and pooled before clustering using a cBot2 followed by 75bp paired-end sequencing on a HiSeq 4000 sequencer (illumina). PDCL normalized RNA expression data is provided in Table S1.

#### Library sequencing

All libraries were sequenced using the Illumina HiSeq 2000/2500 system with TruSeq SBS Kit v3 - HS (200-cycles) reagents (Illumina, Part no. FC-401–3001), to generate paired-end 101 bp reads.

#### Copy number analysis

Matched tumor and normal patient DNA was assayed using Illumina SNP BeadChips as per manufacturer’s instructions (Illumina, San Diego CA) (HumanOmni1-Quad or HumanOmni2.5–8 BeadChips) and analyzed as previously described. PDCL copy number variance is provided in Table S4.

#### Identification and verification of structural variants

The Somatic structural variant pipeline was identified using the qSV tool. A detailed description of its use has been recently published (Nones et al., 2014; Waddell et al., 2015). PDCL mutations are provided in Table S4.

#### Identification of and verification of point mutations

Substitutions and indels were called using a consensus calling approach that included qSNP, GATK and Pindel. The details of call integration and filtering, and verification using orthogonal sequencing and matched sample approaches are as previously described (Nones et al., 2014; Waddell et al., 2015).

#### Mutational signatures

Mutational signatures were defined for genome-wide somatic substitutions, as previously described (Waddell et al., 2015).

#### Metabolite measurements

Steady state metabolomics experiments were performed in cell lines grown to ~80% confluence on 6cm dishes in biological triplicate. Polar and nonpolar metabolites were extracted using Chloroform:Methanol:Water (1:3:1) extraction at 4°. Samples were placed on a rocker for 1hr at 4° then vortex at 4° for 5 minutes, followed by centrifugation at 13,000 g for 3 minutes at 4°. Supernatant was stored at —80° until ready for analysis. Metabolite levels were analyzed by Hydrophilic interaction liquid chromatography (HILIC) on the Dionex UltiMate 3000 RSLC system (ThermoFisher Scientific, Hemel Hemstead, UK) using a ZIC-pHILIC column (150mm x 4.6mm x 5 μM) (Merck). The column was maintained at 30° and samples were eluted with a linear gradient (20mM ammonium carbonate in water, and acetonitrile) over 26 mins at a flow rate of 300uL/min. Instrument .raw files were converted to positive and negative ionisation mode mzXML files. These files were then analyzed using the XCMS/MZMatch/IDEOM pipeline (Creek et al., 2012). PDCL metabolomic measurements are provided in Table S2.

#### Extracellular Metabolic Flux Assays

Measurements of extracellular acidification rate (ECAR) and oxygen consumption rate (OCR) were obtained utilizing the Seahorse XFe96 Analyzer (Seahorse Biosciences) as previously described (Pike Winer and Wu, 2014). In brief, cells were seeded in their respective, fully supplemented medium at a range of densities optimized for each PDCL. 45 minutes prior to starting the assay, cells were equilibrated in seahorse XF DMEM media (Agilent, cat# 103575–100) supplemented with 2mM L-glutamine at 37°C in a non-CO2 incubator. During the assay, indicated compounds were injected into wells at 18-minute intervals. All results were normalized to total cellular protein content per well by RIPA extraction followed quantification with BCA protein assay kit (ThermoFisher Scientific, #23227,) in a 96-well format, with absorbance measured using a Tecan Infinite 200 plate-reader.

#### Glycolysis Stress Test

This assay was initiated in the absence of glucose, with 10 mM glucose, 2.5 μM of Oligomycin (O4876, Sigma-Aldrich) and 50 mM 2-DG (Sigma-Aldrich, #D8375) sequentially added to generate a profile of glycolysis under various conditions, as described previously (Pike Winer and Wu, 2014). PDCL ECAR values after the glycolysis stress test are provided in Table S3. PDCL ECAR values after GSK3βi are provided in Table S6.

#### FAO Assay

This assay functions as an extension to the Mitochondrial Stress Test described by Seahorse Biosciences. In order to stimulate consumption of endogenous fatty acid (FA) reserves, 24-hours prior to beginning this assay, cells were cultured in substrate limited media: DMEM (cat# A1443001) supplemented with 0.5mM glucose, 0.5mM L-carnitine (Sigma-Aldrich, #C0283) and 1% FBS. FAO was quantified as a measurement of OCR upon treatment of cells with either 40 mM FAO inhibitor Etomoxir (Sigma-Aldrich, #E1905) or the FA-palmitate, purchased as Seahorse XF Palmitate-BSA FAO Substrate (Seahorse Biosciences, #102720– 100), as described previously (Pike Winer and Wu, 2014). Initial OCR readings of the assay represent basal levels of respiration in the PDCLs, with sequential additions of 2.5 μM Oligomycin, 1.6 mM CCP (Sigma-Aldrich, #C2759) and a 1 μM combination of Antimycin (Sigma-Aldrich, #A8674) and Rotenone (Sigma-Aldrich, #R8875) providing a profile of OCR under different metabolic conditions. PDCL OCR values after the FAO assay are provided in Table S3.

#### Lactate Production and Glucose Consumption Assays

The L-Lactate content of culture media was measured using the colorimetric-based L-Lactate Assay Kit (Abcam, #ab56331) according to manufacturer’s specifications. 3 × 10^4^ cells were plated in their respective, fully supplemented medium and 24 hours after seeding, this medium was replaced. Cells were cultured for a further 48 hours before medium was taken for analysis. Each test was performed in duplicate, with output adjusted to background lactate levels in medium and normalized to total cell count. Glucose consumption was quantified via the colorimetric-based Glucose Uptake Assay Kit (Abcam, #ab136955) as per the manufacturer’s protocol. Each test was performed in triplicate and normalized to cellular protein content. PDCL lactate production and glucose consumption values are provided in Table S3.

#### *In Vitro* Cytotoxicity assays

Cells were plated in 96-well plates and treated with serial dilutions of indicated inhibitors 24hrs after plating for indicated time points. Cell viability was determined using CellTiter 96® Aqueous non-radioactive cell proliferation assay composed of solutions of a tetrazolium compound [3-(4,5-dimethylthiazol-2-yl)-5-(3-carboxymethoxyphenyl)-2-(4-sulfophenyl)-2H-tetrazolium, inner salt; MTS] and an electron-coupling reagent (phenazine methosulfate; PMS) (Promega, Madison, WI, USA). The assay was performed at an absorbance of 490 nm using an ELISA plate reader (Tecan Trading AG). Background absorbance was corrected for by wells containing medium alone and the absorbance was normalized to 100% (DMSO). 10 technical replicates were performed for 3 independent experiments. IC_50_ calculation and dose response curves were generated using GraphPad Prism 8 (GraphPad Software Inc, La Jolla CA). Normalized cell viability values are provided in Table S6 (GSK3βi single agent) and Table S7 (GSK3βi + ULKi + PORCNi triple treatment).

#### *In situ* hybridization

*In situ* hybridization staining was performed on 4um formalin fixed paraffin embedded sections which had previously been ovened at 600C for 2 hours. *In situ* hybridization detection for WNT7a (401128) and PPIB (313918) (Advanced Cell Diagnostics, Hayward, CA) mRNA was performed using RNAscope 2.5 LS (brown) detection kit (322100; Advanced Cell Diagnostics, Hayward, CA) and performed on a Leica Bond Rx autostainer strictly adhering to the manufacturer’s instructions. WNT7A RNAscope analysis is provided in Table S7.

#### ATACseq library preparation

ATAC-seq libraries were prepared similarly to previously described methods in Buenrostro et al. (2015). A suspension of 100,000 cells were harvested from representative PDCLs and centrifuged for 5 mins at 600 g at 4°C. The cell pellet was washed in 50uL PBS, then centrifuged for 5 mins at 600 g, 4°C. Supernatant was removed and 50uL ATAC-seq cold lysis buffer (10mM Tris-HCl pH 7.4, 10mM NaCl, 3mM MgCl_2_, 0.1% IGEPAL-630) was added to the pellet and gently dislodged. The pellet was immediately centrifuged for 10 mins at 600 g 4°C. The transposition mixture was then made by combining 25uL TD (2X reaction buffer from Nextera kit (Cat#20034197)), 4.7uL TDE1 (Nextera Tn5 Transposase from Nextera kit (Cat#20034197)) and 22.5uL nuclease-free H20. The pellet was then resuspended in the transposition reaction mix and incubated 37°C for 30 mins. Immediately following transposition, the DNA was purified using the QIAGEN MinElute PCR purification kit (Cat# 28004). Eluted transposed DNA was resuspended in 10uL buffer EB. To amplify transposed DNA fragments the following was combined in a 0.2mL PCR tube: 10uL transposed DNA, 10uL nuclease-free H20, 2.5 uL 25uM PCR Primer 1, 2.5 uL 25uM Barcoded PCR primer 2, 25uL NEBNext High-Fidelity 2X PCR master mix (Cat# m0541S). Thermal cycles used were as follows: 1 cycle: 5mins at 2°C, 30 s at 98°C, 5 cycles: 10 s at 98°C, 30 s at 63°C, 1 min at 72°C. To calculate the additional number of cycles required for library amplification a qPCR was performed by combining the following: 5uL of previously PCR-amplified DNA, 4.2 uL H20, 0.4 uL 25uM primer 1, 0.4 uL 25uM primer 2, 5uL 2X SYBR green, 5uL NEB PCR master mix. qPCR thermal cycles used were as following: 1 cycle: 30 s at 98°C, 20 cycles: 10 s at 98°C, 30 s at 63°C, 1 min at 72°C. To calculate additional number of cycles required, plot linear Rn versus cycle and determine the cycle number that corresponds to one-third of the maximum florescent intensity. The remaining 45uL PCR reaction was run the additional cycle number determined by qPCR. Cycle as follows: 1 cycle: 30 s at 98°C, N cycles: 10 s at 98°C, 30 s at 63°C, 1 min at 72°C. Amplified library was purified using QIAGEN Minelute PCR purification kit (Cat# 28004). Library was eluted in 20uL EB buffer. Excess adapters were removed using AMPure XP magnetic beads (Cat# 10136224) and DynaMag-2 magnetic rack. Preliminary library analysis for concentration and size distribution was performed using Agilent High sensitivity DNA kit (Cat# 5067) on the Agilent Bioanalyzer.

#### siRNA screening

Prior to siRNA screening, optimal cell number per well and optimal reverse transfection reagents for each PDCL were identified by assessing transfection efficiency, using six different transfection reagents (Dharmafect 1–4, RNAimax, Lipofectamine 2000), using the manufacturers’ instructions. Experimental conditions were selected that met the following criteria: (i) compared to a mock control (no lipid, no siRNA), the transfection of non-silencing negative control siRNA caused no more than 20% cell inhibition; (ii) compared to non-silencing negative control siRNA, the transfection of PLK1– targeting siRNA caused more than 80% cell inhibition; (iii) cell confluency reached 70% within the range of 4–7 days (Campbell et al., 2016). The later criteria allowed assays to be terminated while cells were in growth phase. Once optimal conditions were established, each PDCL was reverse transfected in a 384 well-plate format with a custom siGENOME siRNA library (Dharmacon, USA) designed to target 714 kinase coding genes, 256 protein phosphatase coding genes, 722 genes implicated in energy metabolism, 73 tumor suppressor genes and 166 genes involved in the repair of DNA damage. Each well in the 384 well-plate arrayed library contained a SMARTpool of four distinct siRNA species targeting different sequences of the target transcript. Each plate was supplemented with non-targeting siCONTROL and siPLK1 siRNAs (Dharmacon, USA). Cell viability was estimated five days after transfection using a luminescent assay detecting cellular ATP levels (CellTiter-Glo, Promega). Luminescence values were processed using the cellHTS2 R package (Boutros et al., 2006). To evaluate the effect of each siRNA pool on cell viability, we log2 transformed the luminescence measurements and then centered these to the median value for each plate. The plate-centered data were scaled to the median absolute deviation (MAD) of the library as a whole to produce robust Z-scores. All screens were performed in triplicate. Screens judged to have poor dynamic range (Z’ factor < 0) (Zhang et al., 1999) or poorly correlated replicates (r < 0.7) were excluded during an evaluation of screen quality. Z scores were adjusted using a quantile normalization (Parrish and Spencer, 2004).

#### Lentiviral transfection

To generate lentiviral particles, 2×10^6^ HEK293FT cells were transfected with a mixture of 2 μg shRNA (see Key Resources Table for shRNA contructs), 0.5 μg pMD2.G (Addgene, Cat#12259) and 1 μg psPAX2 (Addgene, Cat #12260) plasmid DNA using Lipofectamine 2000 (ThermoFisher Scientific, Cat #11668027) as per manufacturers guidelines. Forty-eight hours post transfection, media was removed and filtered through a 0.45 μm Millex-AC filter (Millipore, Cat #SLHV004SL) and mixed at a 1:1 ratio with normal PDCL growth medium, supplemented with polybrene (Millipore, Cat #TR-1003-G) to a final concentration of 5 μg/ml, and added to PDCLs for twenty-four hours. PDCLs were subjected to two rounds of lentiviral infection prior to selection in 2 μg/ml of puromycin (GIBCO, Cat #A1113802).

#### HNF4A and GATA6 siRNA knockdown

For siRNA mediated knockdown experiments, siRNA constructs were purchased from Dharmacon (Key Resources Table) and PDCLs were transfected with 25 pmol siRNA using Lipofectamine RNAiMAX transfection reagent (ThermoFisher Scientific, Cat #13778075) according to manufactures instructions for 6-well format. 72hrs following transfection, PDCLs were analyzed for target knockdown (qRT-PCR and Western Blot analysis) and subjected to RNA-seq or Glycolysis Stress Test analysis. PDCL siHNF4A and siGATA6 RNA-seq, and siHNF4A ECAR values are provided in Table S5.

### QUANTIFICATION AND STATISTICAL ANALYSIS

#### siRNA screen analysis

siRNA “hits” were identified by calculating the median absolute deviation of normalized Z-scores for a given siRNA across all samples and identifying sample Z scores greater than or equal to 2 x the median absolute deviation. This analysis generated a “seed” matrix (n siRNA hits x m samples) which was used as starting input for the Randon Walk with Restart (RWR) algorithm as implemented by the R package dnet (Fang and Gough, 2014). This algorithm was used to identify functionally important subnetworks associated with cell viability from a curated protein-protein interaction network STRING v 10 (Szklarczyk et al., 2015). Considering the complex nature of topological features of human interactome data, we introduce a randomization-based test to evaluate the candidate interactors utilizing 1000 topologically matched random networks. Candidate interactors that remain significant (i.e., p edge < 0.05) were identified and a consensus subnetwork was constructed by collapsing all sample-specific results. The resulting network was visualized using RedeR (Castro et al., 2012). PDCL siRNA screen analysis is provide in Table S6.

#### RNaseq analysis

RNA-seq read mapping was performed by either the bcbio-nextgen RNaseq pipeline (https://bcbio-nextgen.readthedocs.io/en/latest/) or RSEM package (Li and Dewey, 2011). Briefly, after quality control and adaptor trimming, reads were aligned to the GRCh37 genome build using either STAR (Dobin et al., 2013) or RSEM. Count data, obtained from the respective RNaseq pipelines, was normalized using the R/Bioconductor package “DESeq2” to produce rlog transformed expression values. The Combat function from the R package sva was subsequently used to correct for batch effect and to produce an integrated matrix of normalize expression values. This matrix was used for all downstream analyses.

#### WGCNA analysis

Weighted gene co-expression network analysis (WGCNA) was used to generate a transcriptional network from rlog normalized RNa-seq data (Langfelder and Horvath, 2008). Briefly, WGCNA clusters genes into network modules using a topological overlap measure (TOM). The TOM is a highly robust measure of network interconnectedness and essentially provides a measure of the connection strength between two adjacent genes and all other genes in a network. Genes are clustered using 1-TOM as the distance measure and gene modules are defined as branches of the resulting cluster tree using a dynamic branch-cutting algorithm.

The module eigengene is used as a measure of module expression in a given sample and is defined as the first principle component of a module. To relate sample traits of interest to gene modules, sample traits were correlated to module eigengenes and significance determined by a Student asymptotic P value for the given correlations. To relate gene modules to PDCL subtypes, module eigengenes were stratified by subtype and subtype significance determined by Kruskal–Wallis test.

Module preservation as implemented in WGCNA detects the conservation of gene pairs between two networks (e.g., PDCL and bulk). Two composite measures were used to assess module preservation namely, median rank and Zsummary. Median rank was used to identify module preservation and Zsummary to assess significance of module preservation via permutation testing. Permutation was performed 200 times, modules with a Zsummary score > 10 indicate preservation, 2 to 10 indicate weak to moderate preservation and < 2 indicate no preservation in the permutations.

#### Identification of significant subtype specific changes in pathways and/or processes

The R package clipper (Martini et al., 2013) was used to identify pathways and/or processes showing significant change between PDCL subtypes. clipper implements a two-step empirical approach, employing a statistical analysis of means and concentration matrices of graphs derived from pathway topologies, to identify signal paths having the greatest association with a specific phenotype.

#### Methylation analysis

Methylation analysis was performed using Illumina 450K arrays as previously described in (Bailey et al., 2016). Probe filtering, normalization, and differential methylation analysis was performed using the package ‘ChAMP’ (Morris et al., 2014) using default settings. Plots showing regions of differentially methylation were generated using the GVIZ package (Hahne and Ivanek, 2016).

#### ATACseq analysis

Sequencing reads were trimmed and aligned to assembly GRCh38 using bwa mem. Duplicate reads and reads mapping to mitochondrial sequences were subsequently removed. Chromatin accessibility peaks were called using MACS2 (Zhang et al., 2008) and annotated using HOMER (Heinz et al., 2010) and/or ChipSeeker (Yu et al., 2015). Differential accessibility analysis was performed using the R/Bioconductor package DiffBind (Ross-Innes et al., 2012). PDCL ATAC-seq analysis is provided in Table S7.

#### Generation of subtype specific signatures

Pathways and/or processes identified by *clipper* analysis were selected for signature generation. Subtype specific gene signatures representing each pathway and/or process were generated by selecting significant genes in a given graph. Gene weights in each signature represent estimated Z-scores generated from Student t test p values with direction of change provided by the *t test* statistic. The ‘*sig.score*’ function from the R package *genefu* (Haibe-Kains et al., 2012) was used to calculate a specific signature score in a given sample using the signatures generated for each pathway and/or process. PDCL bulk signature scores are provided in Table S1.

#### Gene set enrichment of PDAC subtypes

Gene set enrichment was performed using the R package ‘GSVA’ (Hänzelmann et al., 2013). Gene sets representing PDAC subtypes were generated as previously described (Bailey et al., 2016).

#### Clustering and subtype assignment

The package ‘ConsensusClusterPlus’ (Wilkerson and Hayes, 2010) was used to classify PDCLs according to the expression signatures defined in Moffitt et al. (2015) and Bailey et al. (2016). Gene sets representing PDAC subtypes were generated as previously described. PDCL consensus clustering using Bailey classification (Squamous versus Classical) differential gene expression analysis is provided in Table S1.

#### Pathway analysis

Ontology and pathway enrichment analysis was performed using the R package ‘dnet’ and/or the ClueGO/CluePedia Cytoscape (Bindea et al., 2013; Bindea et al., 2009) plugins as indicated. Visualization and/or generation of network diagrammes was performed using either Cytoscape (Shannon et al., 2003) or the R package *RedeR* (Castro et al., 2012).

#### Plot generation

Heatmaps and oncoplots were generated using the R package *ComplexHeatmap* (Gu et al., 2016). Dotcharts, density plots and box-plots were generated using the R package *ggpubr.* Violin plots were generated using the python package *Seaborn.* Biplot was generated using the R package *ggfortify* (Tang et al., 2016). All other plots were generated using the R package *ggplot2* (Wickham, 2009).

#### Statistical analysis

Statistical parameters are reported in the figures and figure legends. Data are considered significant if p < 0.05. Data are presented as mean ± SD for technical replicates, or mean ± SEM for biological replicates. Data was analyzed using unpaired Student t test when comparing two conditions. One-way ANOVA with Tukey’s multiple comparisons test was performed on comparisons of more than two conditions. Two-way ANOVA was performed on PDCL survival triple inhibitor studies. Kruskal–Wallis test was applied to the indicated stratified scores to determine whether distributions were significantly different. Fisher’s exact tests were used to evaluate the association between dichotomous variables. Survival analysis was performed as previously described (Bailey et al., 2016). Statistical analyses were carried out in either GraphPad Prism 8 (version 8.3.0) or R (version 3.6.1).

### DATA AND CODE AVAILABILITY

Human pancreatic cancer gene expression and genotyping data can be found at the Gene Expression Omnibus Repository (GEO) accession number: GSE36924 and GSE49149. Human pancreatic cancer PDCL alignments, somatic variant calls, annotations and RNA-seq datasets are available at https://dcc.icgc.org/. ATAC-seq sequencing data from patient derived cell lines can be found at BioProject: PRJNA630992. Original data for all datasets in this paper is available at Mendeley Data :https://doi.org/10.17632/74s7crj7xj.1. All software packages used are publicly available through commercial vendors.

## Supplementary Material

Table S1

Table S2

Table S3

Table S4

Table S5

Table S6

Table S7

1

## Figures and Tables

**Table:** KEY RESOURCES TABLE

REAGENT or RESOURCE	SOURCE	IDENTIFIER

Antibodies		

HNF1A (D7Z2Q) Rabbit mAb	Cell signaling Technology	Cat# 89670S; RRID:AB_2728751
HNF4A (C11F12) Rabbit mAb	Cell signaling Technology	Cat# 3113; RRID:AB_2295208
GATA6 Rabbit polyclonal	Abcam	Cat# ab22600; RRID:AB_732529
Phospho-AMPK⍺ (Thr172) (40H9) Rabbit mAb	Cell signaling Technology	Cat# 2535; RRID:AB_331250
GSK-3β (D5C5Z) XP Rabbit mAb	Cell signaling Technology	Cat# 12456; RRID:AB_2636978
DEPTOR (D9F5) Rabbit mAb	Cell signaling Technology	Cat# 11816; RRID:AB_2750575
Hexokinase II (C64G5) mAb	Cell signaling Technology	Cat# 2867; RRID:AB_2232946
AMPK (D63G4) Rabbit mAb	Cell signaling Technology	Cat# 5832; RRID:AB_10624867
Phospho-ULK1 (Ser555) (D1H4) Rabbit mAb	Cell signaling Technology	Cat# 5869; RRID:AB_10707365
Phospho-GSK3B (Ser9) (D85E12) XP Rabbit mAb	Cell signaling Technology	Cat# 5558; RRID:AB_10013750
B-catenin (clone 14) Mouse	BD Biosciences	Cat# 610153; RRID:AB_397554
LKB1 (27D10) Rabbit mAb	Cell signaling Technology	Cat# 3050; RRID:AB_823559
ULK1 (D9D7) Rabbit mAB	Cell signaling Technology	Cat# 6439; RRID:AB_11178933
ATF-3	Cell signaling Technology	Cat# 33593; RRID:AB_2799039
LC3B	Cell signaling Technology	Cat# 2775S; RRID:AB_915950

Bacterial and Virus Strains		

TRC Lentiviral Human HNF4A shRNA, Clone ID TRCN0000019189	Horizon Dharmacon	Cat# RHS3979-201750396
TRC eGFP shRNA positive control	Horizon Dharmacon	Cat #RHS4459
psPAX2 lentiviral packaging plasmid	Addgene	Cat #12260
pMD2.G (VSV-G envelope expressing plasmid)	Addgene	Cat #12259

Biological Samples		

TKCC PDCLs	The TKCC patient derived cell lines were provided by the Australian Pancreatic Cancer Genome Initiative (APGI, https://www.pancreaticcancer.net.au/) and the Garvan Institute of Medical Research (Sydney, Australia).	Hardie et al. (2017). DOI 10.1186/s40170-017-0164-1
PacaDD PDCLs	The PacaDD patient derived cell lines were provided by Universitä tsklinikum Erlangen.	Rückert et al. (2012). https://doi.org/10.1016/j.jss.2011.04.021
HEK293T	American Type Culture Collection	Cat# ATCC CRL-11268

Chemicals, Peptides, and Recombinant Proteins		

6-Aminonicotinamide	USBiological life sciences	Cat# 258294
Dorsomorphin HCl (AMPKi)	Enzo	Cat# ENZ-CHM141-0005
TDZD-8	Sigma	Cat# T8325
D-GLUCOSE (U-13C6, 99%)	Cambridge Isotope Laboratories	Cat# CLM-1396-PK
Tideglusib	Stratec	Cat# B1539-APE
SBI-0206965	Stratec	Cat# S7885-SEL
LGK974	Selleckchem	Cat# S7143
Apo-Transferrin	Sigma-Aldrich	Cat# T1147
D-(+)-Glucose Solution	Sigma-Aldrich	Cat# G8644
DMEM/F12	GIBCO	Cat# 11320-033
Dulbecco’s PBS	GIBCO	Cat# 14190094
EGF Recombinant Human Protein	GIBCO	Cat# PHG0311L
Fetal Bovine Serum (FBS)	GIBCO	Cat# 10270106
Ham’s F12 Nutrient Mixture	GIBCO	Cat# 21765-029
HEPES Buffer Solution	GIBCO	Cat# 15630-049
Hydrocortisone	Sigma-Aldrich	Cat# H0888
IMDM	GIBCO	Cat# 21980-065
Insulin, Human Recombinant	GIBCO	Cat# 12585014
L-Glutamine	GIBCO	Cat# 25030024
Medium M199	GIBCO	Cat# 31150-022
MEM Vitamins	GIBCO	Cat# 11120037
MycoAlert Mycoplasma Detection Kit	Lonza	Cat# LT07-318
O-phosphorylethanolamine	Sigma-Aldrich	Cat# P0503
RPMI 1640 Medium	GIBCO	Cat# 21875034
3,3^’^,5-Triiodo-L-thyronine	Sigma-Aldrich	Cat# T6397
0.5% Trypsin (10X)	GIBCO	Cat# 15400054
2-Deoxy-D-Glucose	Sigma-Aldrich	Cat# D8375
Oligomycin	Sigma-Aldrich	Cat# O4876
Crystal Violet Solution	Sigma-Aldrich	Cat# V5265
Lipofectamine RNAiMAX Transfection Reagent	ThermoFisher Scientific	Cat# 13778075
Lipofectamine 2000 Transfection Reagent	ThermoFisher Scientific	Cat# 11668027
Polybrene Transfection Reagent	Millipore	Cat # TR-1003-G
SYBR Select master Mix	ThermoFisher	Cat# 4472903

Critical Commercial Assays		

CellTiter 96® AQueous Non-Radioactive Cell Proliferation Assay (MTS)	Promega	Cat# G5430
Pierce BCA Protein Assay Kit	Thermo Scientific	Cat# 23227
Pierce ECL Western Blotting Substrate	Thermo Scientific	Cat# 32106
Seahorse XF Palmitate-BSA FAO Substrate	Agilent	Cat# 102720-100
L-Lactate Assay Kit	Abcam	Cat# ab56331
Glucose Uptake Assay Kit	Abcam	Cat# ab136955
Seahorse XF glycolysis stress test kit	Aglient	Cat# 103030-100
Seahorse XFe96 FluxPaks	Agilent	Cat# 102416-100
RNeasy Mini Kit	QIAGEN	Cat# 74104
Illumina Tagment DNA TDE1 Enzyme and Buffer kit	Illumina	Cat# 20034197
MinElute PCR Purification Kit	QIAGEN	Cat# 28004
NEBNext High-Fidelity 2X PCR Master Mix	New England Biolabs	Cat# M0541S
Agencourt AMPure XP beads	fisher scientific	Cat# 10136224
Bioanalyzer DNA analysis	Agilent	Cat# 5067
AffinityScript Multiple temperature cDNA synthesis kit	Agilent Technologies	Cat# 200436

Deposited Data		

Human Pancreatic Cancer DNA and RNA-seq data	Bailey et al. (2016). https://doi.org/10.1038/nature16965	European Genome-phenome Archive (EGA): accession code EGAS00001000154
Human Pacreatic Cancer gene expression and genotyping data	Bailey et al. (2016). https://doi.org/10.1038/nature16965	NCBI Gene Expression Omnibus (GEO) under accession codes GSE49149 and GSE36924
Human Pancreatic Cancer PDCL ATAC-seq sequencing data	This paper	BioProject: PRJNA630992
Tables S1, S2, S3, S4, S5, S6, and S7	This paper	https://dx.doi.org/10.17632/74s7crj7xj.1

Experimental Models: Organisms/Strains		

Pdx1-Cre, LSL-Kras^G12D^	Hingorani et al. (2005)	DOI:10.1016/j.ccr.2005.04.023
Pten_fl_ and LSL-Trp53^R172H^	Kennedy et al. (2011)	DOI:10.1016/j.molcel.2011.02.020

Oligonucleotides		

ON-TARGETplus Non-targeting Pool/siRNA #1	Dharmacon	Cat# D-001810
ON-TARGETplus HNF4A SMARTpool siRNA	Dharmacon	Cat# L-003406-00
Hs_GAPDH_1_SG QuantiTect Primer	QIAGEN	Cat# QT00079247
Hs_WNT7A_1_SG QuantiTect Primer	QIAGEN	Cat# QT00012278
Hs_LGR5_1_SG QuantiTect Primer Assay	QIAGEN	Cat# QT00027720
Hs_AXIN2_1_SG QuantiTect Primer Assay	QIAGEN	Cat# QT00037639
ON-TARGETplus Human HNF4A (3172) siRNA - Individual	horizon	Cat# J-003406-08-0002
ON-TARGETplus Human HNF4A (3172) siRNA - Individual	horizon	Cat# J-003406-09-0002
ON-TARGETplus Human GATA6 SMARTpool siRNA	Dharmacon	Cat# L-008351-00-0005

Software and Algorithms		

Dnet	Hai Fang and Julian Gough	https://cran.r-project.org/web/packages/dnet/index.html
ClueGo Cytoscape	Bindea et al., 2009	http://apps.cytoscape.org/apps/cluego
CluePedia Cytoscape	Bindea et al., 2013	http://apps.cytoscape.org/apps/cluepedia
RedeR	Castro et al., 2012	https://bioconductor.org/packages/release/bioc/html/RedeR.html
MACS2	Zhang et al., 2008	https://taoliu.github.io/MACS/
Cytoscape	Shannon et al., 2003	https://cytoscape.org/
ComplexHeatmap	Gu et al., 2016	https://bioconductor.org/packages/release/bioc/html/ComplexHeatmap.html
Ggpubr	CRAN	https://cran.r-project.org/web/packages/ggpubr/index.html
Seaborn	python	https://seaborn.pydata.org/
Ggfortify	Tang et al., 2016	https://cran.r-project.org/web/packages/ggfortify/index.html
ggplot2	Wickham, 2009	https://cran.r-project.org/web/packages/ggplot2/index.html
qSV	omicX	https://omictools.com/qsv-tool
qSNP	omicX	https://omictools.com/qsnp-tool
GATK	Broad Institute	https://gatk.broadinstitute.org/hc/en-us
Pindel	Sanger	https://github.com/genome/pindel
HOMER	Heinz et al., 2010	http://homer.ucsd.edu/homer/
STRING	Szklarczyk et al., 2015	https://string-db.org/cgi/input.pl
ChipSeeker	Yu et al., 2015	https://bioconductor.org/packages/release/bioc/html/ChIPseeker.html
RSEM	Li and Dewey, 2011	https://deweylab.github.io/RSEM/
STAR	Dobin et al., 2013	https://code.google.com/archive/p/rna-star/
ChAMP	Morris et al., 2014	https://bioconductor.org/packages/release/bioc/html/ChAMP.html
Gviz	Hahne and Ivanek, 2016	https://bioconductor.org/packages/release/bioc/html/Gviz.html
DiffBind	Ross-Innes et al., 2012	https://bioconductor.org/packages/release/bioc/html/DiffBind.html
Clipper	Martini et al., 2020	https://bioconductor.org/packages/release/bioc/html/clipper.html
Genefu////	Gendoo et al., 2020	https://www.bioconductor.org/packages/release/bioc/html/genefu.html
GSVA	Hänzelmann et al., 2013	https://bioconductor.org/packages/release/bioc/html/GSVA.html
ConsensusClusterPlus	Wilkerson and Hayes, 2010	http://bioconductor.org/packages/release/bioc/html/ConsensusClusterPlus.html

## Highlights

- HNF4A loss upregulates GSK3β and drives a squamous-like metabolic profile
- GSK3β targeting inhibits glycolysis in squamous patient-derived cell lines (PDCLs)
- A subset of squamous PDCLs acquires GSK3β drug tolerance
- ATAC-seq analysis reveals an accessible WNT gene program in drug-tolerant PDCLs
