# Supplementary material for: HNF4A and GATA6 Loss Reveals Therapeutically Actionable Subtypes in Pancreatic Cancer
Source: Cell Rep. Author manuscript; Available in PMC 2022 Sep 26. (PMC9511995; doi:10.1016/j.celrep.2020.107625)
Supplement: 1 [file NIHMS1625804-supplement-1.pdf]

Cell Reports, Volume *31*

Supplemental Information

HNF4A and *GATA6* Loss Reveals Therapeutically Actionable Subtypes in Pancreatic Cancer

Holly Brunton, Giuseppina Caligiuri, Richard Cunningham, Rosie Upstill-Goddard, Ulla-Maja Bailey, Ian M. Garner, Craig Nourse, Stephan Dreyer, Marc Jones, Kim Moran-Jones, Derek W. Wright, Viola Paulus-Hock, Colin Nixon, Gemma Thomson, Nigel B. Jamieson, Grant A. McGregor, Lisa Evers, Colin J. McKay, Aditi Gulati, Rachel Brough, Ilirjana Bajrami, Stephen J. Pettitt, Michele L. Dziubinski, Simon T. Barry, Robert Grützmann, Robert Brown, Edward Curry, Glasgow Precision Oncology Laboratory, Australian Pancreatic Cancer Genome Initiative, Marina Pajic, Elizabeth A. Musgrove, Gloria M. Petersen, Emma Shanks, Alan Ashworth, Howard C. Crawford, Diane M. Simeone, Fieke E.M. Froeling, Christopher J. Lord, Debabrata Mukhopadhyay, Christian Pilarsky, Sean E. Grimmond, Jennifer P. Morton, Owen J. Sansom, David K. Chang, Peter J. Bailey, and Andrew V. Biankin

Figure S1

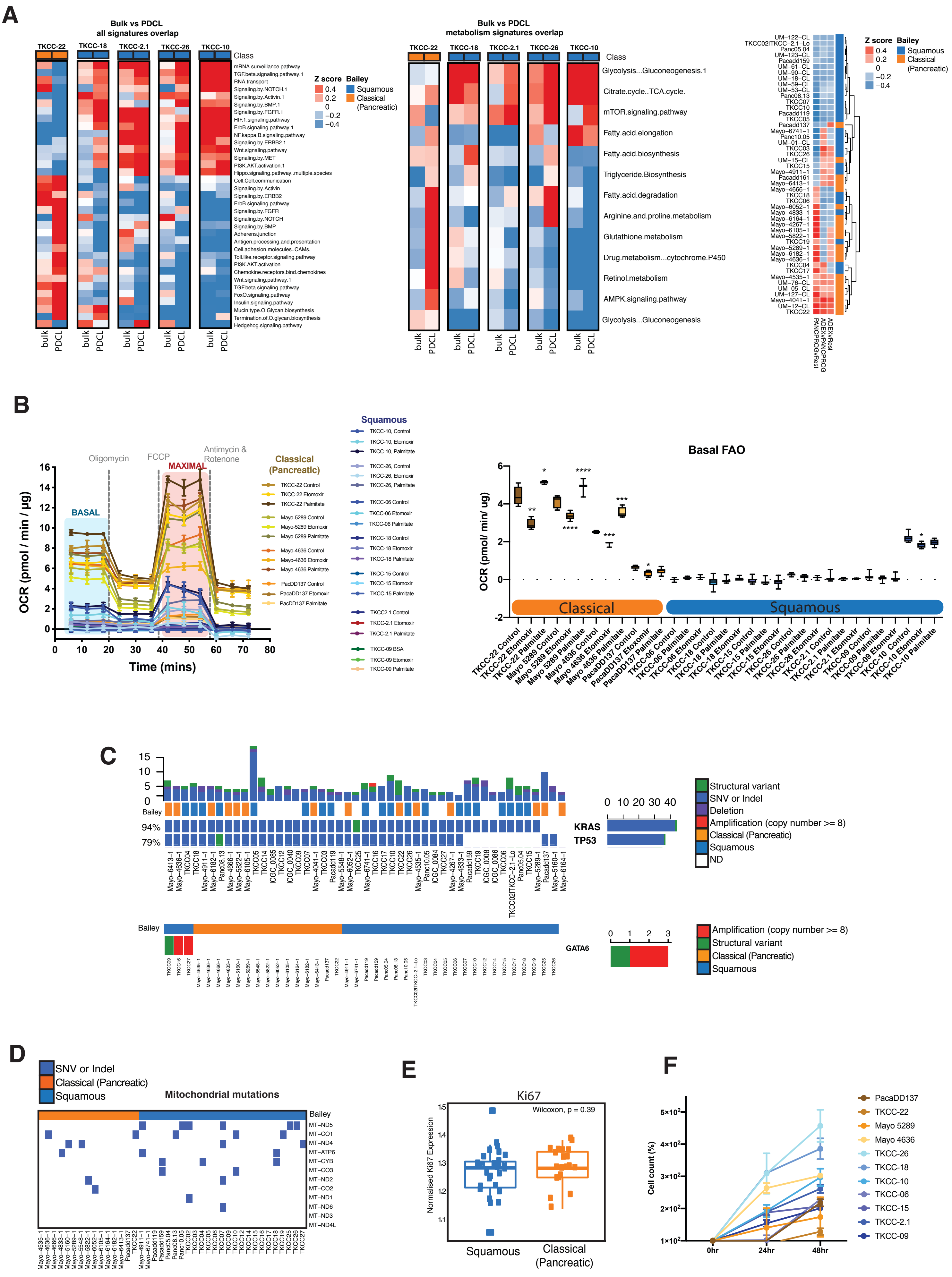
A
Bulk vs PDCL
all signatures overlap
TKCC-22
TKCC-18
TKCC-2.1
TKCC-26
TKCC-10
Class
Z score
Bailey
Squamous
Classical
(Pancreatic)
bulk
PDCL
Bulk vs PDCL
metabolism signatures overlap
Glycolysis...Gluconeogenesis.1
Citrate.cycle..TCA.cycle.
mTOR.signaling.pathway
Fatty.acid.elongation
Fatty.acid.biosynthesis
Triglyceride.Biosynthesis
Fatty.acid.degradation
Arginine.and.proline.metabolism
Glutathione.metabolism
Drug.metabolism...cytochrome.P450
Retinol.metabolism
AMPK.signaling.pathway
Glycolysis...Gluconeogenesis
B
Oligomycin
FCCP
Antimycin &
Rotenone
MAXIMAL
BASAL
OCR (pmol / min / µg)
Time (mins)
Classical
(Pancreatic)
Squamous
Basal FAO
OCR (pmol/ min/ ug)
Classical
Squamous
C
Bailey
KRAS
TP53
Structural variant
SNV or Indel
Deletion
Amplification (copy number >= 8)
Classical (Pancreatic)
Squamous
ND
GATA6
D
SNV or Indel
Classical (Pancreatic)
Squamous
Mitochondrial mutations
E
Ki67
Wilcoxon, p = 0.39
Normalised Ki67 Expression
Squamous
Classical
(Pancreatic)
F
Cell count (%)
0hr
24hr
48hr
PacaDD137
TKCC-22
Mayo 5289
Mayo 4636
TKCC-26
TKCC-18
TKCC-10
TKCC-06
TKCC-15
TKCC-2.1
TKCC-09

Figure S1. Related to Figure 1: Squamous PDCLs preferentially utilise glycolysis as their main energy source independent of KRAS or mitochondrial mutational status.

(**A**) Direct comparison of matched bulk tumour and PDCL samples for gene signatures significantly altered in PDCLs. **(B)** (left) Agilent Seahorse XF Cell Mito Stress Test profiles of Squamous and Classical PDCLs treated with BSA, palmitate or etomoxir. (right) OCR values for cells treated as in (B) corrected for oxygen consumption resultant from processes other than mitochondrial respiration. Box plots are annotated using one-way ANOVA. Error bars represent mean $\pm$ SD. Independent experiments are shown, n=>6. **(C)** Oncoplot of *KRAS* and *TP53* (top) and GATA6 somatic mutations in PDCLs (below). Structural variations (green), non-silent mutations (blue), deletion (purple) and amplification ≥ 8 copies (red). **(D)** Mitochondria mutational status across the PDCLs. (**E)** Ki67 expression across Classical (Pancreatic) and Squamous PDCLs from RNA-seq analysis. Classical (Pancreatic) = orange/brown; Squamous = blue. Wilcoxon text. **(F)** PDCL proliferation analysed by cell count.

Figure S2

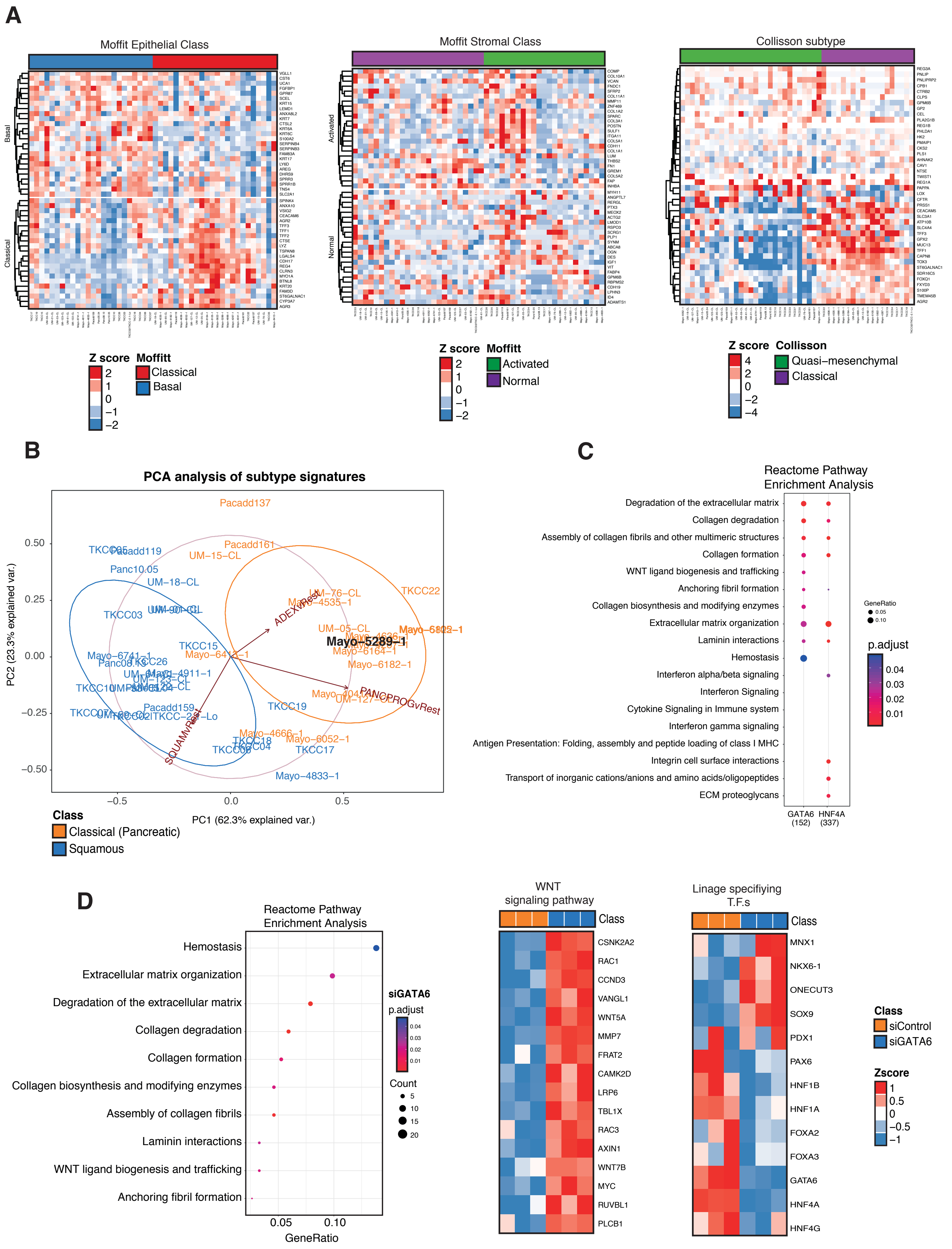
A
Moffit Epithelial Class
Basal
Classical
Moffit Stromal Class
Activated
Normal
Collisson subtype
Z score
Moffitt
Classical
Basal
Activated
Normal
Collisson
Quasi–mesenchymal
Classical
B
PCA analysis of subtype signatures
PC2 (23.3% explained var.)
PC1 (62.3% explained var.)
Pacadd137
Pacadd161
UM–15–CL
Panc10.05
UM–18–CL
TKCC03
TKCC22
UM–76–CL
Mayo–4535–1
ADEXvRest
UM–05–CL
Mayo–5289–1
Mayo–6182–1
Mayo–6164–1
TKCC15
Mayo–6741–1
TKCC26
UM–123–CL
Pacadd159
PANCPROGvRest
SQUAMvRest
TKCC19
Mayo–4666–1
Mayo–6052–1
TKCC18
TKCC04
TKCC17
Mayo–4833–1
Class
Classical (Pancreatic)
Squamous
C
Reactome Pathway Enrichment Analysis
Degradation of the extracellular matrix
Collagen degradation
Assembly of collagen fibrils and other multimeric structures
Collagen formation
WNT ligand biogenesis and trafficking
Anchoring fibril formation
Collagen biosynthesis and modifying enzymes
Extracellular matrix organization
Laminin interactions
Hemostasis
Interferon alpha/beta signaling
Interferon Signaling
Cytokine Signaling in Immune system
Interferon gamma signaling
Antigen Presentation: Folding, assembly and peptide loading of class I MHC
Integrin cell surface interactions
Transport of inorganic cations/anions and amino acids/oligopeptides
ECM proteoglycans
GATA6 (152)
HNF4A (337)
GeneRatio
p.adjust
D
Reactome Pathway Enrichment Analysis
Hemostasis
Extracellular matrix organization
Degradation of the extracellular matrix
Collagen degradation
Collagen formation
Collagen biosynthesis and modifying enzymes
Assembly of collagen fibrils
Laminin interactions
WNT ligand biogenesis and trafficking
Anchoring fibril formation
GeneRatio
siGATA6
p.adjust
Count
WNT signaling pathway
Class
CSNK2A2
RAC1
CCND3
VANGL1
WNT5A
MMP7
FRAT2
CAMK2D
LRP6
TBL1X
RAC3
AXIN1
WNT7B
MYC
RUVBL1
PLCB1
Linage specifiying T.F.s
Class
MNX1
NKX6-1
ONECUT3
SOX9
PDX1
PAX6
HNF1B
HNF1A
FOXA2
FOXA3
GATA6
HNF4A
HNF4G
Class
siControl
siGATA6
Zscore

Figure S2. Related to Figure 2: *GATA6* knock down activates distinct gene programmes associated with the Squamous subtype. (A) Unsupervised clustering of PDCLs using Moffit epithelial and stromal and Collisson classifications. **(B)** Principal components analysis RNA-seq data from PDAC PDCLs showing separation into Classical (Pancreatic), ADEX and Squamous subtypes. Mayo 5289 is highlighted in bold **(C)** Reactome pathway enrichment analysis of significantly altered pathways identified after *GATA6 or HNF4A* knock down in Mayo 5289 PDCL. Adjusted P value for each annotation is represented by colour scale. Gene ratio is represented by dot size. **(D)** Reactome pathway enrichment analysis (left) and heatmap of gene expression changes (right) after *GATA6* knockdown from RNA-seq analysis in Mayo 5289 PDCL. Enriched terms and pathways were identified as significant at an adjusted P value ≤ 0.05 and FDR ≤ 0.05.

Figure S3

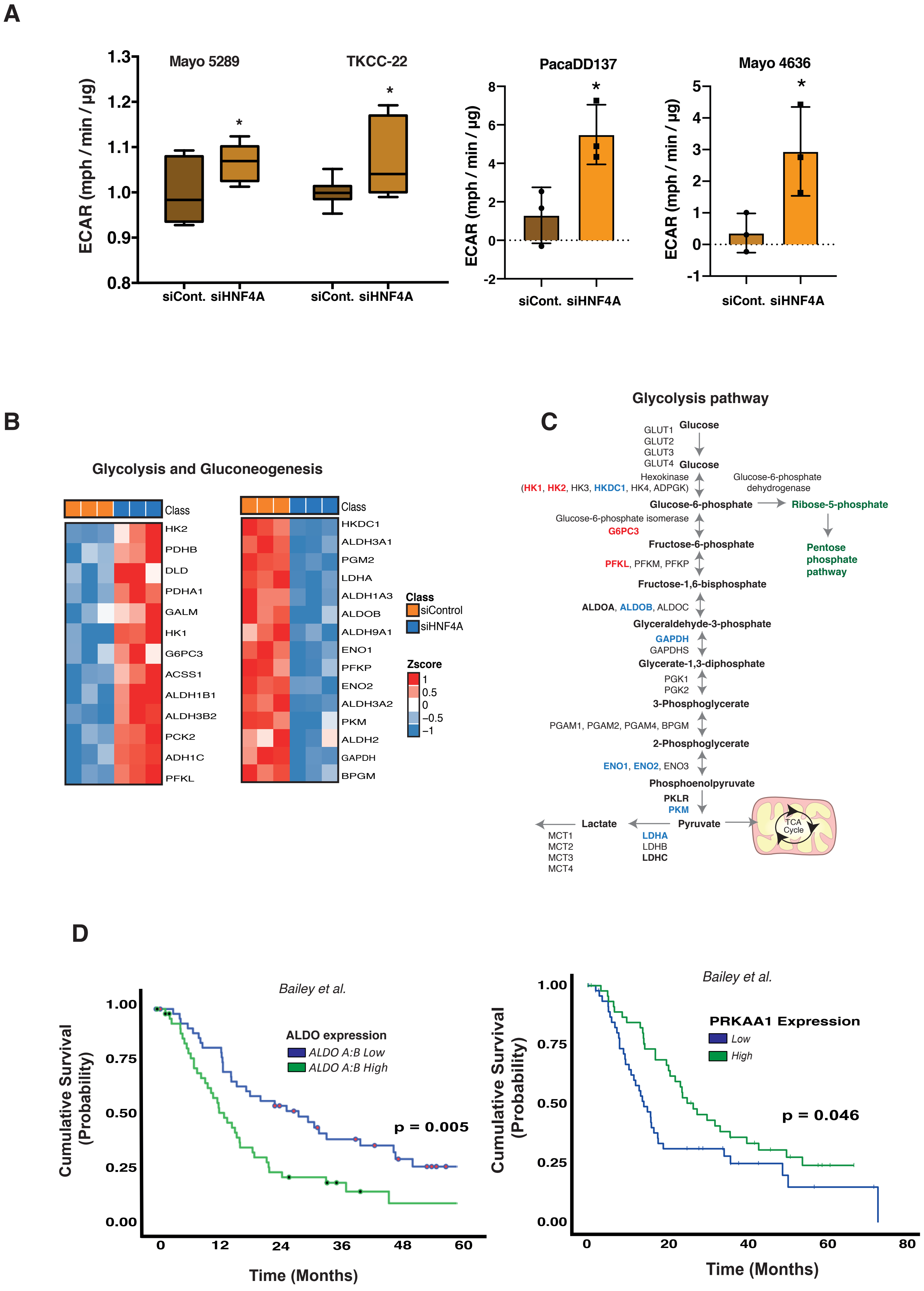
A
Mayo 5289
TKCC-22
PacaDD137
Mayo 4636
ECAR (mph / min / µg)
siCont. siHNF4A
B
Glycolysis and Gluconeogenesis
Class
HK2
PDHB
DLD
PDHA1
GALM
HK1
G6PC3
ACSS1
ALDH1B1
ALDH3B2
PCK2
ADH1C
PFKL
HKDC1
ALDH3A1
PGM2
LDHA
ALDH1A3
ALDOB
ALDH9A1
ENO1
PFKP
ENO2
ALDH3A2
PKM
ALDH2
GAPDH
BPGM
siControl
siHNF4A
Zscore
C
Glycolysis pathway
Glucose
GLUT1
GLUT2
GLUT3
GLUT4
Hexokinase
(HK1, HK2, HK3, HKDC1, HK4, ADPGK)
Glucose-6-phosphate dehydrogenase
Glucose-6-phosphate
Ribose-5-phosphate
Pentose phosphate pathway
Glucose-6-phosphate isomerase
G6PC3
Fructose-6-phosphate
PFKL, PFKM, PFKP
Fructose-1,6-bisphosphate
ALDOA, ALDOB, ALDOC
Glyceraldehyde-3-phosphate
GAPDH
GAPDHS
Glycerate-1,3-diphosphate
PGK1
PGK2
3-Phosphoglycerate
PGAM1, PGAM2, PGAM4, BPGM
2-Phosphoglycerate
ENO1, ENO2, ENO3
Phosphoenolpyruvate
PKLR
PKM
Lactate
Pyruvate
TCA Cycle
MCT1
MCT2
MCT3
MCT4
LDHA
LDHB
LDHC
D
Bailey et al.
ALDO expression
ALDO A:B Low
ALDO A:B High
p = 0.005
Cumulative Survival (Probability)
Time (Months)
PRKAA1 Expression
Low
High
p = 0.046

Figure S3. Related to Figure 3: Gene expression changes after *HNF4A* knockdown are reflective of PDAC patient survival. (A) Heatmap of significantly altered glycolysis and gluconeogenesis genes after *HNF4A* knockdown in Classical (Pancreatic) Mayo 5289 PDCLs. Orange = Mayo 5289 siControl; blue = Mayo 5289 siHNF4A **(B)** Schema of the glycolysis pathway, with enzymes whose mRNA expression was upregulated after *HNF4*A knockdown in red and down regulated in blue. **(C)** ECAR in Classical (Pancreatic) PDCLs following siRNA mediated knockdown of *HNF4A*. Box plots are annotated using one-way ANOVA, mean $\pm$ SD. Technical replicates are shown, n >= 6. For all graphs: *p = <0.05. **(D)** Correlation graph demonstrating a trend for low HNF4A expression with high GSK3B expression in the squamous subtype from bulk tumour samples described in Bailey et al., 2016 (left) and in the PDCLs (right). **(E)** Kaplan-Meier plots showing overall survival based on data reported by Bailey et al., 2016. Tumour samples were stratified based on *PRKAA1* (right) expression or a high or low *ALDOA:B* ratio (left). logrank P value.

Figure S4

A

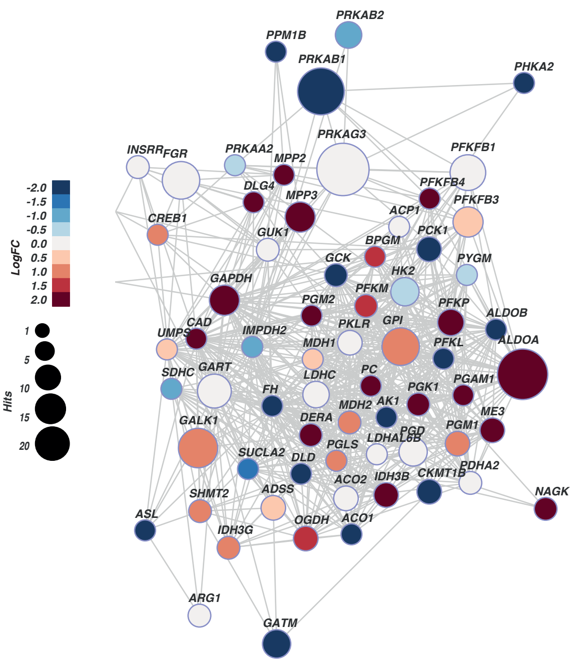

B

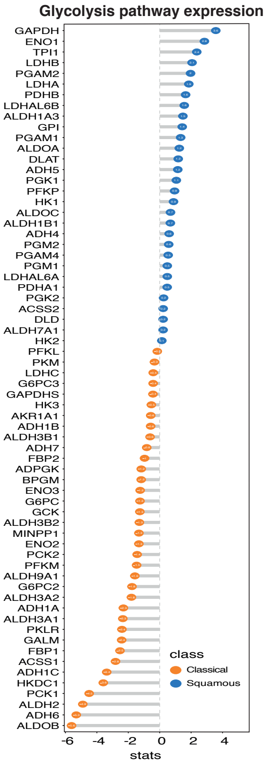

C

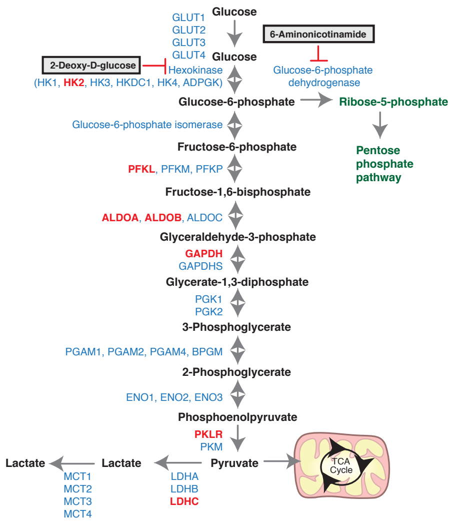

siRNA screen hits

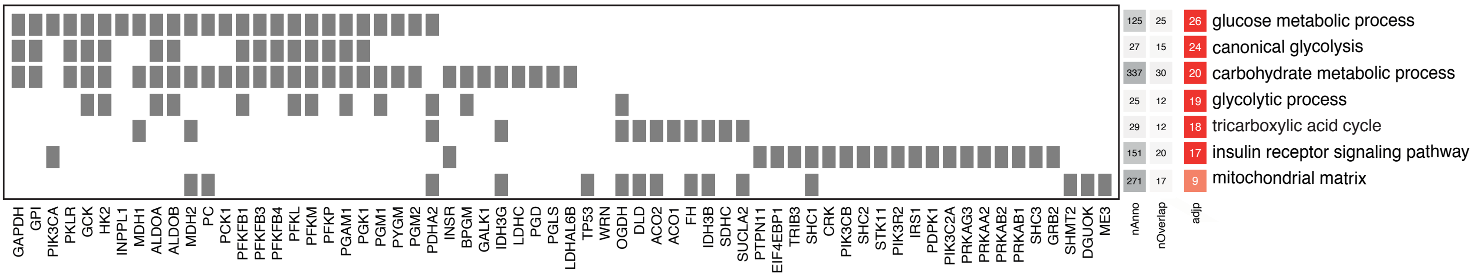

D

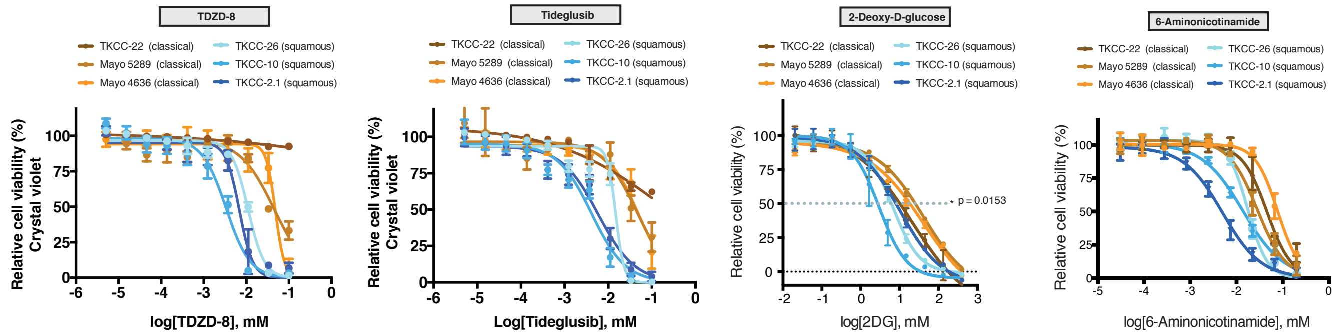

Figure S4. Related to Figure 4: siRNA screen identifies metabolic dependencies in PDAC PDCLs. (A) Functional interaction (F1) sub-network of siRNA screen hits in PDAC PDCLs. Different node colour represents subtype expression for each gene, with orange representing Classical (Pancreatic) and blue Squamous, and the size of each node is relative to the number of siRNA hits. **(B)** Differential expression of glycolysis genes between subtypes based on RNA-seq analysis. **(C)** Schema of the glycolysis pathway, with enzymes that were identified as a positive hit from the siRNA screen in red. **(D)** Cell viability dose response curves for GSK3β inhibitors (TDZD-8 and Tideglusib), the glucose analogue 2-Deoxy-D-glucose and the pentose phosphate pathway inhibitor, 6-aminonicotinamide.

Figure S5

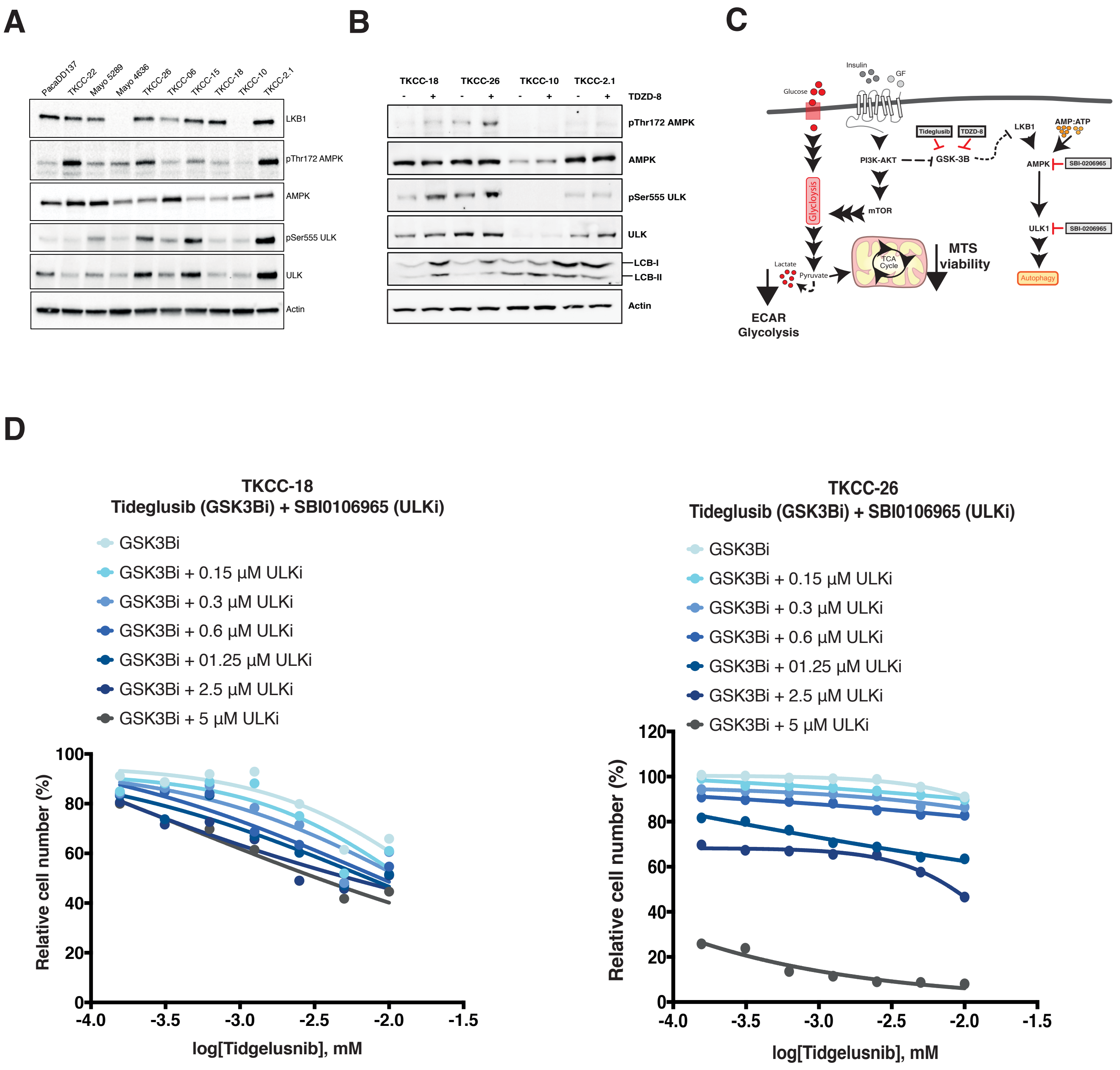
A
PacaDD137
TKCC-22
Mayo 5289
Mayo 4636
TKCC-26
TKCC-06
TKCC-15
TKCC-18
TKCC-10
TKCC-2.1
LKB1
pThr172 AMPK
AMPK
pSer555 ULK
ULK
Actin
B
TKCC-18
TKCC-26
TKCC-10
TKCC-2.1
TDZD-8
pThr172 AMPK
AMPK
pSer555 ULK
ULK
LCB-I
LCB-II
Actin
C
Insulin
GF
Glucose
Glycolysis
PI3K-AKT
mTOR
Tideglusib
TDZD-8
GSK-3B
LKB1
AMP:ATP
AMPK
SBI-0206965
ULK1
Autophagy
TCA Cycle
MTS viability
Lactate
Pyruvate
ECAR
Glycolysis
D
TKCC-18
Tideglusib (GSK3Bi) + SBI0106965 (ULKi)
GSK3Bi
GSK3Bi + 0.15 μM ULKi
GSK3Bi + 0.3 μM ULKi
GSK3Bi + 0.6 μM ULKi
GSK3Bi + 01.25 μM ULKi
GSK3Bi + 2.5 μM ULKi
GSK3Bi + 5 μM ULKi
Relative cell number (%)
log[Tidgelusnib], mM
TKCC-26
Tideglusib (GSK3Bi) + SBI0106965 (ULKi)
GSK3Bi
GSK3Bi + 0.15 μM ULKi
GSK3Bi + 0.3 μM ULKi
GSK3Bi + 0.6 μM ULKi
GSK3Bi + 01.25 μM ULKi
GSK3Bi + 2.5 μM ULKi
GSK3Bi + 5 μM ULKi
Relative cell number (%)
log[Tidgelusnib], mM

Figure S5. Related to Figure 4: Combinational targeting of AMPK and ULK is unable to resensitize PDCLs to GSK3β inhibition. **(A)** Protein expression of the LKB1-AMPK-ULK signalling axis across representative PDAC PDCLs under untreated conditions. 20μg of the same protein lysate was probed with stated antibodies on different blots. Actin panel is a representative loading control (ULK loading shown). (B) Markers for autophagy induction (ULK and LC3) tested by WB in indicated PDCLs treated with TDZD-8 for 144hr **(C)** Model for potential resistance mechanisms after extended GSK3β inhibition which demonstrates the points of therapy intervention used in this study. **(D)** Squamous PDCLs treated with a two-fold dilution range (beginning at 10uM) of GSK3β inhibitor Tideglusib plus indicated concentration of AMPK/ULK inhibitor SBI 0206965 for 144hrs. Proliferation was assessed using nuclear cell count.

Figure S6

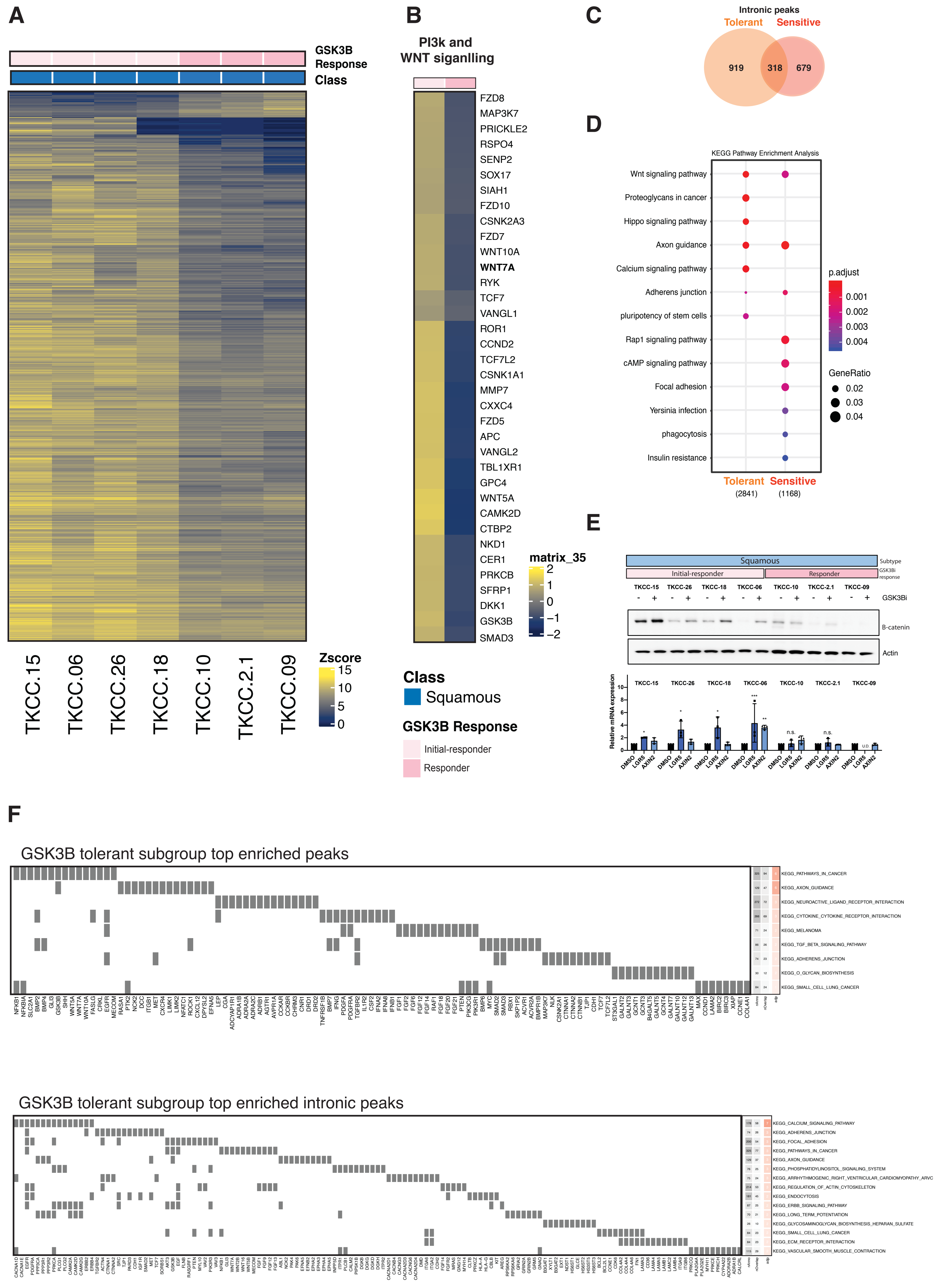

A
GSK3B Response
Class
TKCC.15
TKCC.06
TKCC.26
TKCC.18
TKCC.10
TKCC.2.1
TKCC.09
Zscore
15
10
5
0
B
PI3k and WNT siganlling
FZD8
MAP3K7
PRICKLE2
RSPO4
SENP2
SOX17
SIAH1
FZD10
CSNK2A3
FZD7
WNT10A
WNT7A
RYK
TCF7
VANGL1
ROR1
CCND2
TCF7L2
CSNK1A1
MMP7
CXXC4
FZD5
APC
VANGL2
TBL1XR1
GPC4
WNT5A
CAMK2D
CTBP2
NKD1
CER1
PRKCB
SFRP1
DKK1
GSK3B
SMAD3
matrix_35
2
1
0
−1
−2
Class
Squamous
GSK3B Response
Initial-responder
Responder
C
Intronic peaks
Tolerant
Sensitive
919
318
679
D
KEGG Pathway Enrichment Analysis
Wnt signaling pathway
Proteoglycans in cancer
Hippo signaling pathway
Axon guidance
Calcium signaling pathway
Adherens junction
pluripotency of stem cells
Rap1 signaling pathway
cAMP signaling pathway
Focal adhesion
Yersinia infection
phagocytosis
Insulin resistance
p.adjust
0.001
0.002
0.003
0.004
GeneRatio
0.02
0.03
0.04
Tolerant (2841)
Sensitive (1168)
E
Squamous
Subtype
Initial-responder
Responder
GSK3Bi response
TKCC-15
TKCC-26
TKCC-18
TKCC-06
TKCC-10
TKCC-2.1
TKCC-09
GSK3Bi
B-catenin
Actin
Relative mRNA expression
DMSO
LGR5
AXIN2
n.s.
U.D.
F
GSK3B tolerant subgroup top enriched peaks
KEGG_PATHWAYS_IN_CANCER
KEGG_AXON_GUIDANCE
KEGG_NEUROACTIVE_LIGAND_RECEPTOR_INTERACTION
KEGG_CYTOKINE_CYTOKINE_RECEPTOR_INTERACTION
KEGG_MELANOMA
KEGG_TGF_BETA_SIGNALING_PATHWAY
KEGG_ADHERENS_JUNCTION
KEGG_O_GLYCAN_BIOSYNTHESIS
KEGG_SMALL_CELL_LUNG_CANCER
GSK3B tolerant subgroup top enriched intronic peaks
KEGG_CALCIUM_SIGNALING_PATHWAY
KEGG_ADHERENS_JUNCTION
KEGG_FOCAL_ADHESION
KEGG_PATHWAYS_IN_CANCER
KEGG_AXON_GUIDANCE
KEGG_PHOSPHATIDYLINOSITOL_SIGNALING_SYSTEM
KEGG_REGULATION_OF_ACTIN_CYTOSKELETON
KEGG_ENDOCYTOSIS
KEGG_ERBB_SIGNALING_PATHWAY
KEGG_LONG_TERM_POTENTIATION
KEGG_GLYCOSAMINOGLYCAN_BIOSYNTHESIS_HEPARAN_SULFATE
KEGG_SMALL_CELL_LUNG_CANCER
KEGG_ECM_RECEPTOR_INTERACTION
KEGG_VASCULAR_SMOOTH_MUSCLE_CONTRACTION

Figure S6. Related to Figure 6: The Squamous subtype separates into two distinct chromatin subgroups with unique chromatin accessibility and promoter usage. (A) ATAC-seq density plots of accessible genes in 7 squamous PDCLs sub-grouped based on response to GSK3βi. **(B)** KEGG pathway enrichment analysis of enriched intronic peaks for genes involved PI3K and WNT signalling identified in the GSK3Bi tolerant Squamous subgroup. **(C)** Venn diagram showing number of unique genes assigned to intronic peaks between GSk3βi tolerant and sensitive squamous PDCLs from ATAC-seq analysis. **(D)** KEGG pathway enrichment analysis of unique genes assigned to all peaks between GSk3Bi tolerant and sensitive squamous PDCLs. **(E)** Markers for WNT signalling tested by qPCR (LGR5 and AXIN2) and WB (b-cateinin) in squamous PDCLs treated with GSK3βI for 144hr. **(F)** KEGG pathway enrichment analysis of top enriched peaks and top enriched intronic peaks identified in the GSK3Bi tolerant Squamous subgroup.

Figure S7

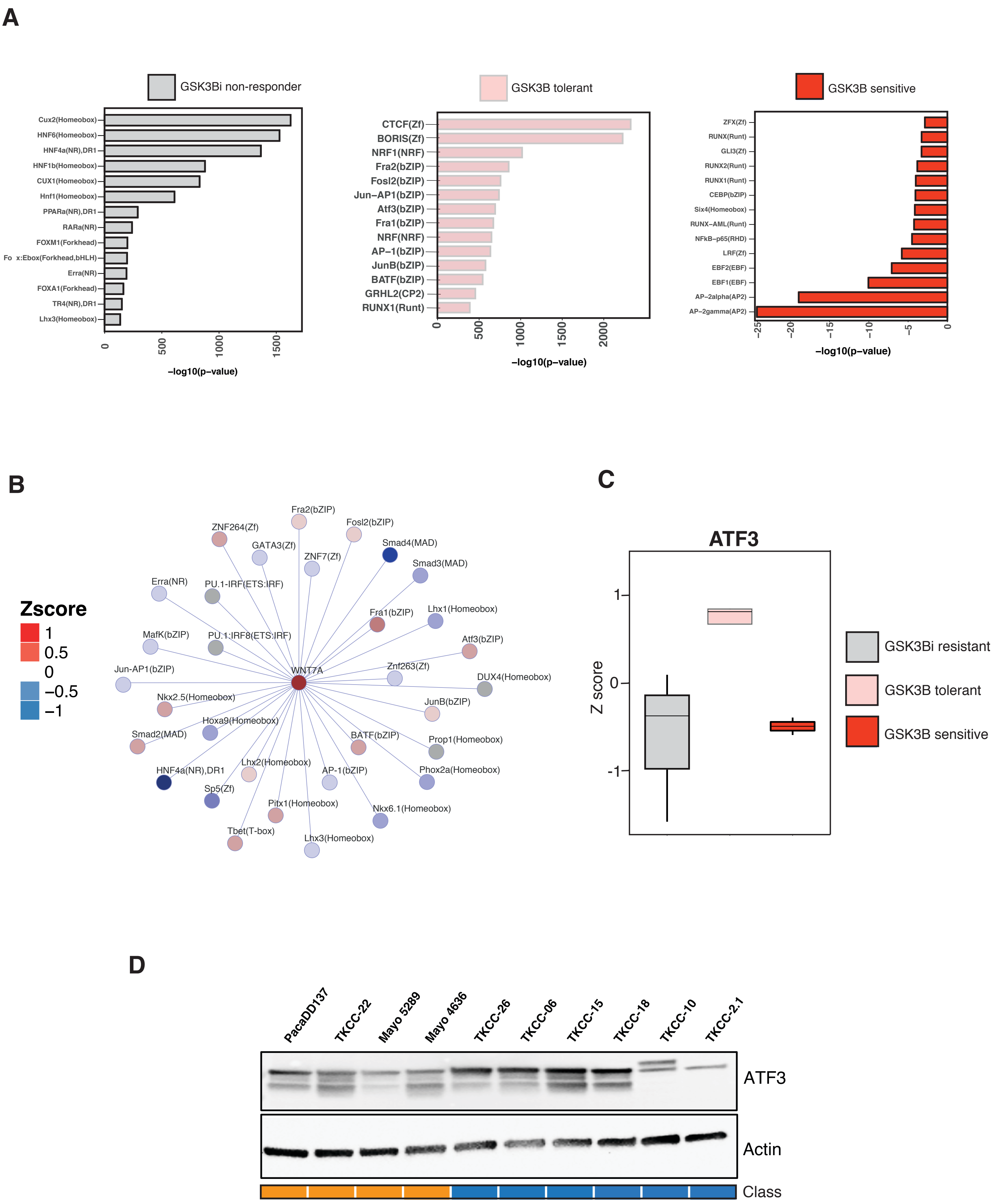
A
GSK3Bi non-responder
Cux2(Homeobox)
HNF6(Homeobox)
HNF4a(NR),DR1
HNF1b(Homeobox)
CUX1(Homeobox)
Hnf1(Homeobox)
PPARa(NR),DR1
RARa(NR)
FOXM1(Forkhead)
Erra(NR)
FOXA1(Forkhead)
TR4(NR),DR1
Lhx3(Homeobox)
0
500
1000
1500
−log10(p−value)
GSK3B tolerant
CTCF(Zf)
BORIS(Zf)
NRF1(NRF)
Fra2(bZIP)
Fosl2(bZIP)
Jun−AP1(bZIP)
Atf3(bZIP)
Fra1(bZIP)
NRF(NRF)
AP−1(bZIP)
JunB(bZIP)
BATF(bZIP)
GRHL2(CP2)
RUNX1(Runt)
0
500
1000
1500
2000
−log10(p−value)
GSK3B sensitive
ZFX(Zf)
RUNX(Runt)
GLI3(Zf)
RUNX2(Runt)
RUNX1(Runt)
CEBP(bZIP)
Six4(Homeobox)
RUNX−AML(Runt)
NFkB−p65(RHD)
LRF(Zf)
EBF2(EBF)
EBF1(EBF)
AP−2alpha(AP2)
AP−2gamma(AP2)
−25
−20
−15
−10
−5
0
−log10(p−value)
B
Zscore
1
0.5
0
−0.5
−1
Fra2(bZIP)
ZNF264(Zf)
Fosl2(bZIP)
GATA3(Zf)
Smad4(MAD)
ZNF7(Zf)
Smad3(MAD)
Erra(NR)
PU.1-IRF(ETS:IRF)
Fra1(bZIP)
Lhx1(Homeobox)
MafK(bZIP)
PU.1:IRF8(ETS:IRF)
Atf3(bZIP)
Jun-AP1(bZIP)
WNT7A
Znf263(Zf)
DUX4(Homeobox)
Nkx2.5(Homeobox)
JunB(bZIP)
Hoxa9(Homeobox)
Smad2(MAD)
BATF(bZIP)
Prop1(Homeobox)
HNF4a(NR),DR1
Lhx2(Homeobox)
AP-1(bZIP)
Phox2a(Homeobox)
Sp5(Zf)
Pitx1(Homeobox)
Nkx6.1(Homeobox)
Tbet(T-box)
Lhx3(Homeobox)
C
ATF3
Z score
1
0
-1
GSK3Bi resistant
GSK3B tolerant
GSK3B sensitive
D
PacaDD137
TKCC-22
Mayo 5289
Mayo 4636
TKCC-26
TKCC-06
TKCC-15
TKCC-18
TKCC-10
TKCC-2.1
ATF3
Actin
Class

Figure S7. Related to Figure 6: Transcription factor motif analysis identifies ATF3 as a potential regulator of *WNT7A* in HNF4A^{low}/GATA6^{low} Squamous PDCL subgroup. (A) Transcription factor motifs significantly enriched in HNF4A^{high}/GATA6^{high}, HNF4A^{low}/GATA6^{low}, and HNF4A^{low}/GATA6^{high} PDCL subgroups identified from ATCA-seq analysis. **(B)** Node diagram showing known transcription factors for *WNT7A*. Nodes are coloured according to mRNA expression (Z score) of each gene expression in Squamous PDCLs. **(C)** Box plot showing expression of *ATF3* from RNA-seq data of PDAC PDCLs. **(D)** Immunoblots of the TF ATF3 across representative PDAC PDCLs from the Classical (Pancreatic) and Squamous subtypes.
